# Nitrate exposure reprograms hepatic amino acid and nutrient sensing pathways prior to exercise: A metabolomic and transcriptomic investigation in zebrafish *(Danio rerio)*

**DOI:** 10.3389/fmolb.2022.903130

**Published:** 2022-07-19

**Authors:** Rosa M. Keller, Laura M. Beaver, Mary C. Prater, Lisa Truong, Robyn L. Tanguay, Jan F. Stevens, Norman G. Hord

**Affiliations:** ^1^ University of California, San Francisco, San Francisco, CA, United States; ^2^ Linus Pauling Institute, Oregon State University, Corvallis, OR, United States; ^3^ Department of Foods and Nutrition, College of Family and Consumer Sciences, University of Georgia, Athens, GA, United States; ^4^ Sinnhuber Aquatic Research Laboratory and the Department of Environmental and Molecular Toxicology, Oregon State University, Corvallis, OR, United States; ^5^ College of Pharmacy, Oregon State University, Corvallis, OR, United States; ^6^ OU Health, Harold Hamm Diabetes Center, Department of Nutritional Sciences, College of Allied Health, University of Oklahoma Health Sciences Center, Oklahoma City, OK, United States

**Keywords:** nitrate, zebrafish, metabolomics (OMICS), energy metabolism, liver

## Abstract

**Scope:** Nitrate supplementation is a popular ergogenic aid that improves exercise performance by reducing oxygen consumption during exercise. We investigated the effect of nitrate exposure and exercise on metabolic pathways in zebrafish liver.

**Materials and methods:** Fish were exposed to sodium nitrate (606.9 mg/L), or control water, for 21 days and analyzed at intervals during an exercise test. We utilized untargeted liquid chromatography-tandem mass spectrometry (LC-MS/MS) analysis and measured gene expression of 24 genes central to energy metabolism and redox signaling.

**Results:** We observed a greater abundance of metabolites involved in endogenous nitric oxide (NO) metabolism and amino acid metabolism in nitrate-treated liver at rest, compared to rested controls. In the absence of exercise, nitrate treatment upregulated expression of genes central to nutrient sensing (*pgc1a*)*,* protein synthesis (*mtor*) and purine metabolism (*pnp5a and ampd1*) and downregulated expression of genes involved in mitochondrial fat oxidation (*acaca and cpt2*).

**Conclusion:** Our data support a role for sub-chronic nitrate treatment in the improvement of exercise performance, in part, by improving NO bioavailability, sparing arginine, and modulating hepatic gluconeogenesis and glycolytic capacity in the liver.

## Introduction

Dietary nitrate improves exercise performance by reducing the oxygen cost of exercise and improving the efficiency of ATP resynthesis in mitochondria, however the mechanisms for this effect are not well understood (Larsen et al., 2011; Affourtit et al., 2015). We demonstrated a dose-dependent effect of nitrate on exercise performance in zebrafish in two previously published studies (Axton et al., 2019; Keller et al., 2021). Using untargeted metabolomics technologies of whole zebrafish and zebrafish muscle, we attributed these nitrate-induced, performance-enhancing effects to changes in the metabolic programming of muscle prior to exercise by increasing the availability of energy-producing metabolites, including phosphocreatine and ATP, required for exercise. Nitrate supplementation is acknowledged as a performance-enhancing dietary component by an expert committee appointed by the International Olympic Committee (Maughan et al., 2018).

In addition to its performance-enhancing effect, dietary nitrate is shown to have protective effects against a milieu of cardiometabolic diseases including cardiovascular disease, fatty liver disease, type 2 diabetes, and metabolic syndrome (McNally et al., 2016; Lundberg et al., 2018; Cordero-Herrera et al., 2019). The effect of nitrate treatment is primarily attributed to its reduction to nitrite (NO_2_^−^) and nitric oxide (NO), termed the nitrate-nitrite-nitric oxide pathway (Govoni et al., 2008). NO is a ubiquitous signaling molecule responsible for regulating vasodilation and blood flow by stimulating cyclic guanosine monophosphate (cGMP)-dependent vasodilation (Gewaltig and Kojda, 2002). NO is produced endogenously through metabolism of the amino acid L-arginine and O_2_ to L-citrulline in the vascular endothelium via endothelial nitric oxide synthase (eNOS). NO stimulates uptake and oxidation of glucose and fatty acids in liver, heart, adipose and skeletal muscle tissue (Higaki et al., 2001; Dai et al., 2013). Both exercise and nitrate treatment are known to have beneficial effects on liver function in adults with fatty liver disease (Liu et al., 2021). Yet little is known about the effect of nitrate on liver metabolism (Ahlborg et al., 1974; Hu et al., 2020). It is of interest to determine the effect of nitrate on endogenous NO metabolism and energy metabolism in metabolically active tissues such as the liver.

The liver is a primary storage organ of nitrate and uptake of nitrate in liver is increased with nitrate supplementation in rodents (Gilliard et al., 2018), making it a relevant organ to investigate the impact of nitrate exposure on liver metabolism. The liver plays a crucial role in energy homeostasis during exercise by regulating hepatic uptake, release of glucose, and oxidation of triglycerides to be exported to skeletal muscle (Trefts et al., 2015). Amino acids are a major fuel for the liver and their oxidative conversion to glucose accounts for about half of daily oxygen consumption of the liver (Jungas et al., 1992). Most amino acids are converted to glucose, via gluconeogenesis, and this allows the liver to make nearly two thirds of the total energy available from oxidation of amino acids accessible to peripheral tissues, especially skeletal muscle during exercise (Jungas et al., 1992). Generally, fish have higher dietary requirements for protein and lower requirements for fat and carbohydrate than humans (Jia et al., 2017). A recent study measured the contribution of amino acids, carbohydrates and fatty acids to provide ATP in liver and skeletal muscle in zebrafish using stable isotope tracers (Jia et al., 2017). Glutamate, glutamine, alanine and leucine were shown to provide ∼80% of ATP production in liver and skeletal muscle (Jia et al., 2017). Furthermore, a newly developed technique to measure oxidation of fuels showed that 6 h post-feeding, amino acids were preferentially metabolized for energy production (Ferreira et al., 2019). In fed fish, the respiratory quotient increased from 0.89 to 0.97, whereas the nitrogen quotient increased from 0.072 to 0.140, representing ∼52% amino acid/protein usage (Ferreira et al., 2019). Taken together, these studies demonstrate a greater need for amino acid availability to produce ATP.

To our knowledge, this is the first study to investigate the effect of nitrate treatment on whole liver metabolism using untargeted metabolomics. Our previous experimental analysis in whole zebrafish showed that 21 days of nitrate exposure reduced the oxygen cost of exercise, similar to humans (Axton et al., 2019). Metabolomics analysis in whole zebrafish and zebrafish muscle revealed that this exercise performance effect was coincident with increased availability of metabolite fuels (i.e., ATP, glycolytic and tricarboxylic acid (TCA) intermediates, lactate and ketone bodies) in rested zebrafish (Axton et al., 2019; Keller et al., 2021). These findings in whole fish were striking and led us to investigate organ-specific changes in metabolism specifically in the liver. Here, we sought to survey potential metabolic and genomic determinants for the effect of nitrate exposure and exercise on liver metabolism using an untargeted discovery-based approach. Zebrafish have become increasingly popular as a model organism to study developmental biology, as the development and function of zebrafish organs are strikingly similar to humans and have been used to study liver disease, cardiovascular disease, muscle disease and cancer (Pham et al., 2017). Furthermore, the molecular regulation of liver development is largely conserved between zebrafish and mammals (Pham et al., 2017). Due to the importance of the concerted action between skeletal muscle and liver during acute exercise, we aimed to determine the effect of nitrate exposure on liver metabolism, with and without exercise, using untargeted metabolomics. Liver gene expression data of rested zebrafish treated with nitrate or controls were analyzed to find a potential overlap between exercise-regulated metabolites and relevant targets in the hepatic transcriptome.

## Methods and materials

### Zebrafish husbandry

Wild type zebrafish (5D) were raised and maintained at the Sinnhuber Aquatic Research Laboratory (SARL) at Oregon State University on standard lab diet (Gemma Micro. Skretting, Tooele, France) in accordance with protocols approved by the OSU Institutional Animal Care and Use Committee (ACUP# 5087). Adult fish were maintained at six fish per tank (3 male and 3 female) in 4-L of aerated water in metal tanks. Experiments were conducted in several cohorts of healthy adult fish, fish from each cohort were equally distributed between all treatment groups. A total of 124 male and female zebrafish were exposed to 606.9 mg/L sodium nitrate, or control water, for 21 days as described previously (Axton et al., 2019). The fish water was replaced every 36–42 h to maintain low ammonia concentrations and consistent nitrate treatments, monitored for pH (6.8–7), total ammonia concentrations (0–2.0 ppm), and temperature (27–29°C). Nitrate was dissolved in freshly prepared fish water and, unless otherwise indicated, chemicals were purchased from Sigma-Aldrich (St. Louis, MO). Fish were exercised in a strenuous, graded exercise test as previously described (Axton et al., 2019). The rested condition constituted zebrafish that never entered the swim tunnel post-treatment. Peak exercise condition was directly after the 20-min swim at the highest speed (40 cm/second) and a total of 130 min of swimming in the tunnel. The post exercise period is defined as the period immediately after the completion of the assay where fish returned to 5 cm/second for 20 min. The six experimental conditions analyzed were named as follows for treatment and exercise state: 1) control-rest, 2) control-peak exercise, 3) control-post exercise, 4) nitrate-rest, 5) nitrate-peak exercise, and 6) nitrate-post exercise. Fish were humanely euthanized with an overdose of the anesthesia drug tricaine mesylate, and all efforts were made to minimize suffering. A subset of fish used for gene expression analysis were humanely euthanized with rapid colling, followed by cervical dislocation (Wallace et al., 2018). Fish were then dried, weighed, measured for standard length, and liver were collected and snap frozen in liquid nitrogen. Samples were stored in −80°C until used for gene expression and metabolomics analysis.

### Extraction of zebrafish liver for analysis

Eighteen livers per treatment group were snap frozen using liquid nitrogen after 3 weeks of treatment and two livers were pooled together for each sample (*n* = 9/treatment group). Each sample was added into 2 ml pre-filled tubes containing 300 mg of RNAse and DNAse free zirconium oxide beads (0.5 mm diameter, ceria stabilized, Next Advance, Averill Park, NY). A mixture of 80:20 methanol: water at −80°C was used as the extraction solvent as previously described (Choi et al., 2015). Liver samples were homogenized with a bullet blender (Precellys1 24-bead-based homogenizer three times for 30 s at 6500 rpm). Extracts were incubated at −20°C for 1 h and then centrifuged at 13,000 rpm (Eppendorf, Hauppauge, NY). The supernatant was split into two 1.5 ml Eppendorf tubes: 200 μL was aliquoted for untargeted metabolomics analysis, and the remainder (variable volume) was reserved and stored at −80°C. The residual liver solids were then freeze-dried and re-suspended in solvent (1 mg tissue/50 μl 80:20 methanol:water) and 10ul CUDA internal standard. Extracts were sonicated for 5 min, clarified by centrifugation (13,000 × *g*, 10 min) and supernatants transferred to mass spectrometry vials.

### LC-MS/MS untargeted metabolomics

Liquid chromatography tandem mass spectrometry (LC-MS/MS) -based untargeted metabolomics was performed in both negative and positive ion modes, as previously described (Axton et al., 2019; Garcia-Jaramillo et al., 2020). Briefly, high pressure liquid chromatography (HPLC) was performed on a Shimadzu Nexera system (Shimadzu, Columbia, MD) with a phenyl-3 stationary phase column (Inertsil Phenyl-3, 2.1 mm × 150 mm, GL Sciences, Torrance, CA) coupled to a quadrupole time-of-flight mass spectrometer (AB SCIEX TripleTOF 5600). The flow rate was 0.1200 ml/min, and mobile phases were composed of water (A) and methanol (B), both with 0.1% formic acid. The auto-sampler temperature was held at 15°C, the column oven temperature at 50°C, and the injection volume was 1 μl. Time-of-flight (TOF) mass spectrometry (MS) was operated with an acquisition time of 0.25 s and a scan range of 70–1,200 Da. Each MS/MS scan had an accumulation time of 0.1 s and a range of 80–1,200 Da using information-dependent MS/MS acquisition (IDA). The mass calibration was automatically performed every 6 injections using an APCI positive/negative calibration solution (AB SCIEX) via a calibration delivery system (CDS). A separate quality control (QC) pool sample was prepared by combining 10 μl of each sample. Quality of sample run was assured by: 1) randomization of the sequence of samples in which they were analyzed, 2) injection of QC samples every 10 samples, 3) auto-calibration performed every 2 samples. All samples were analyzed in time-of-flight (TOF) scan mode in both positive and negative ion mode. MS/MS analysis (IDA and SWATH^®^) were performed on QC samples.

### LC-MS/MS data processing

LC-MS/MS data was analyzed with PeakView with XIC Manager 1.2.0 (AB SCIEX, Framingham, MA) and Progenesis QI (Waters Corporation, Newcastle, UK) software. Data were evaluated based on the accurate mass similarity, isotope similarity, and fragmentation score. Supporting Information (Supplementary Table S1, DOI: 10.6084/m9.figshare.19287542 URL: https://figshare.com/s/616b1258de1ad40ffc74) lists identified (L1) and putatively assigned metabolites (L2 annotations), and provides access to the following properties: formula, retention time, monoisotopic ion mass, adducts, mass error, and molecular formula. Level 1 (L1) and level 2 (L2) metabolite annotations were assigned based on level of confidence of annotations as described previously (Sumner et al., 2007; Alcazar Magana et al., 2020). L1 annotations were determined using PeakView by matching accurate mass (error <10 ppm), retention time (error <10%), MS/MS fragmentation (library score >70), and isotope distribution (error <20%) with an in-house library of 650 commercially available standards (including IROA Technology, Bolton, MA). L2 metabolite identities were assigned using Progenesis QI software that queries METLIN, Human Metabolome Database, ChemSpider, and LipidBlast databases. Peak lists from PeakView were exported to MultiQuant 4.0.2 (SCIEX) to integrate chromatograms to obtain peak areas. Samples were normalized to the peak abundance of the internal standard.

### Gene expression

Expression of genes related to glucose metabolism, lipid metabolism, redox signaling and nutrient signaling were evaluated after 21 days of treatment in liver by quantitative real-time PCR (Supplementary Table S2, DOI: 10.6084/m9.figshare.19287560, URL: https://figshare.com/s/c658383947c487bfe713). Whole liver were homogenized in 700 μl of Trizol reagent (ThermoFisher) with 0.5 mm zirconium oxide beads in a bullet blender (Next Advance, Averill Park, NY). RNA was purified using with Direct-zol RNA MiniPrep kit (Zymo Research, Irvine, CA) following manufactures recommendations. cDNA was synthesized using 2 μg of total RNA and SuperScript IV First-Strand Synthesis SuperMix (ThermoFisher Scientific, Waltham, MA). Expression of 24 genes were performed using real-time quantitative PCR (7,900 Fast Real-Time PCR System, Applied Biosystems) as previously described (Beaver et al., 2017). Final gene expression was calculated using the 2^−ΔΔCT method, relative to the level of the housekeeping genes selenoprotein F (*selenof*) or ribosomal protein L13a (*rpl13a*). In order to normalize and control for changes in gene expression, the copy number of the gene of interest was divided by the copy number of the housekeeping gene and then expressed relative to the mean level found in control samples. 

### Statistical analysis

For untargeted metabolomics analyses, annotated metabolites were used to conduct multivariate statistical analysis. Pathway analysis and partial least squares-discriminant analysis (PLS-DA) and Variable Importance in Projection (VIP) scores were generated with MetaboAnalyst 5.0. The significance of individual metabolites between the treatment groups was assessed with a one-way ANOVA followed by Fisher’s post-hoc analysis and Holm FDR-correction, with a *p*-value of <0.05 and a *q*-value < 0.1 indicating significance. If needed, data were logarithmically transformed to correct for unequal variance or non-normal distribution. Gene expression data was assessed using individual non-parametric Wilcoxon rank tests using GraphPad Prism (La Jolla, CA). Figures were generated with Prism 8 (GraphPad Software, San Diego, CA), PowerPoint 2016 (Microsoft, Redmond, WA), and MetaboAnalyst 5.0.

## Results

### Untargeted metabolomics results

**FIGURE 1:**
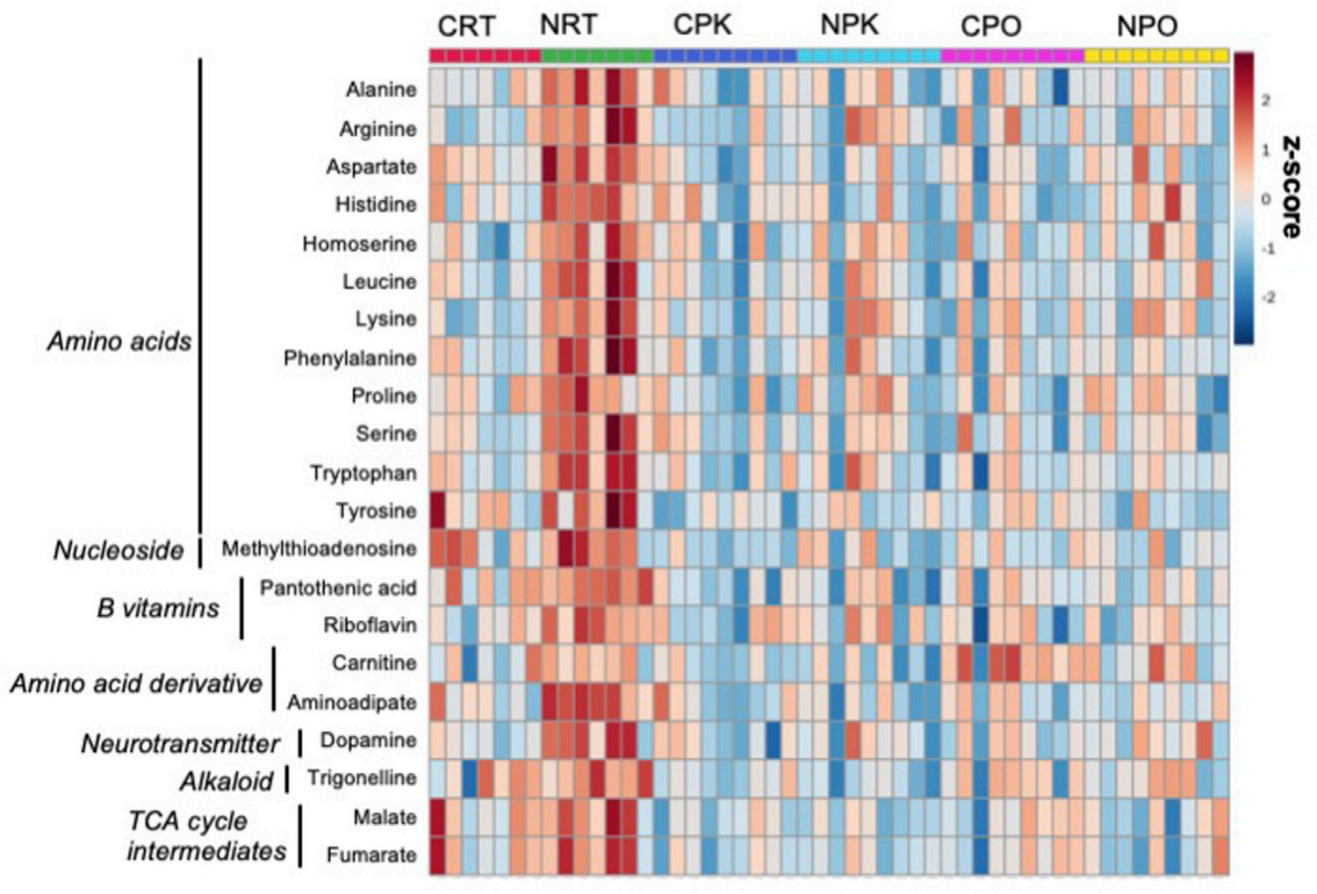
Nitrate treatment altered abundance of metabolites at rest, compared to control liver at rest. The metabolites were chosen based on top FDR-corrected *p*-values and physiological significance. Colors indicate z-score (standard deviation from the mean). The heat map was generated with MetaboAnalyst 5.0 using normalized data (log transformation, auto-scaling) using Euclidean distance measure (n = 7–9/group).

Untargeted metabolomics was performed using LC-MS/MS analysis and 157 metabolites were annotated (Supplementary Table S1). Of these, 31 metabolites were significantly changed among at least one treatment group (Supplementary Table S3, DOI: 10.6084/m9.figshare.19287569, URL: https://figshare.com/s/b3f74b8bd32c56b04395). Peak abundance of annotated metabolites is provided in Supplementary Table S4 (DOI : 10.6084/ m9.figshare.19287572, URL: https://figshare.com/s/4e04511dc8e02064ba12. The PLS-DA plot demonstrates technical separation between control liver and nitrate-treated liver at rest and clustering of quality control samples (Supplementary Figure S5 (DOI : 10.6084/ m9.figshare.19967969, URL: https://figshare.com/s/d901d5a287aa96fe413a). Relative standard deviation of the internal standard was 28.6% for QC samples and 69.6% for biological samples. The majority of significant changes were independent of exercise and were found between control and nitrate-treated liver at rest. A heatmap illustrates the relative number of metabolites significantly changed in at least one treatment group that were relevant to energy metabolism. A pattern of greater abundance of metabolites with nitrate treatment was observed at rest compared to control liver at rest (Figure 1). Nitrate treatment significantly increased amino acids, B vitamins (pantothenic acid and riboflavin), dopamine, trigonelline and TCA cycle intermediates in rested liver, relative to control liver.

### NO homeostasis

**FIGURE 2:**
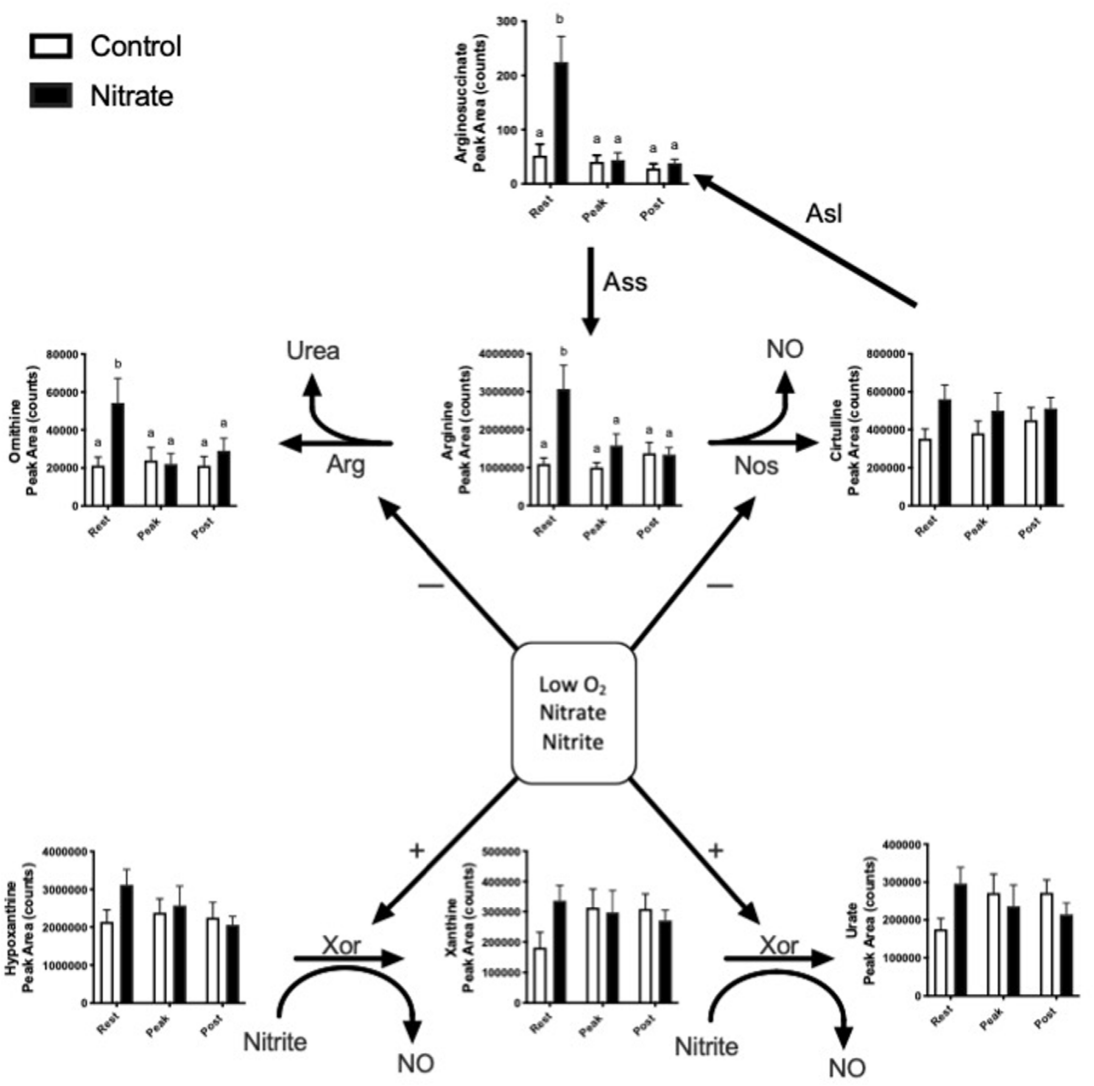
Metabolites related to NO homeostasis in zebrafish. Relative levels of metabolites metabolized by nitric oxide synthase (NOS), arginase (ARG) and xanthine oxidoreductase (XOR). Citrulline can also be recycled back to arginine. Asl, argininosuccinate lyase; Ass, argininosuccinate synthase. Labeled means without a common letter differ. (One-way ANOVA with Fisher’s post-hoc and Holm FDR-correction, *p* < 0.1 indicating significance, *n* = 7–9/group).

Nitrate is known to alter endogenous NO synthesis and arginine bioavailability. Therefore, we next aimed to determine the effect of nitrate on NO homeostasis in the liver (Figure 2). We observed an increase in arginine, ornithine and arginosuccinate metabolite levels by 2.5–5.1-fold in nitrate-treated liver at rest, compared to rested controls, and these metabolites in nitrate-treated liver returned to control levels with exercise. No significant difference in arginine, ornithine or arginosuccinate was observed in nitrate-treated liver at peak exercise or post exercise conditions relative to control liver at the same exercise condition. No statistically significant increase in hypoxanthine, xanthine or urate was observed at rest in any treatment group.

### Amino acid metabolism

**FIGURE 3:**
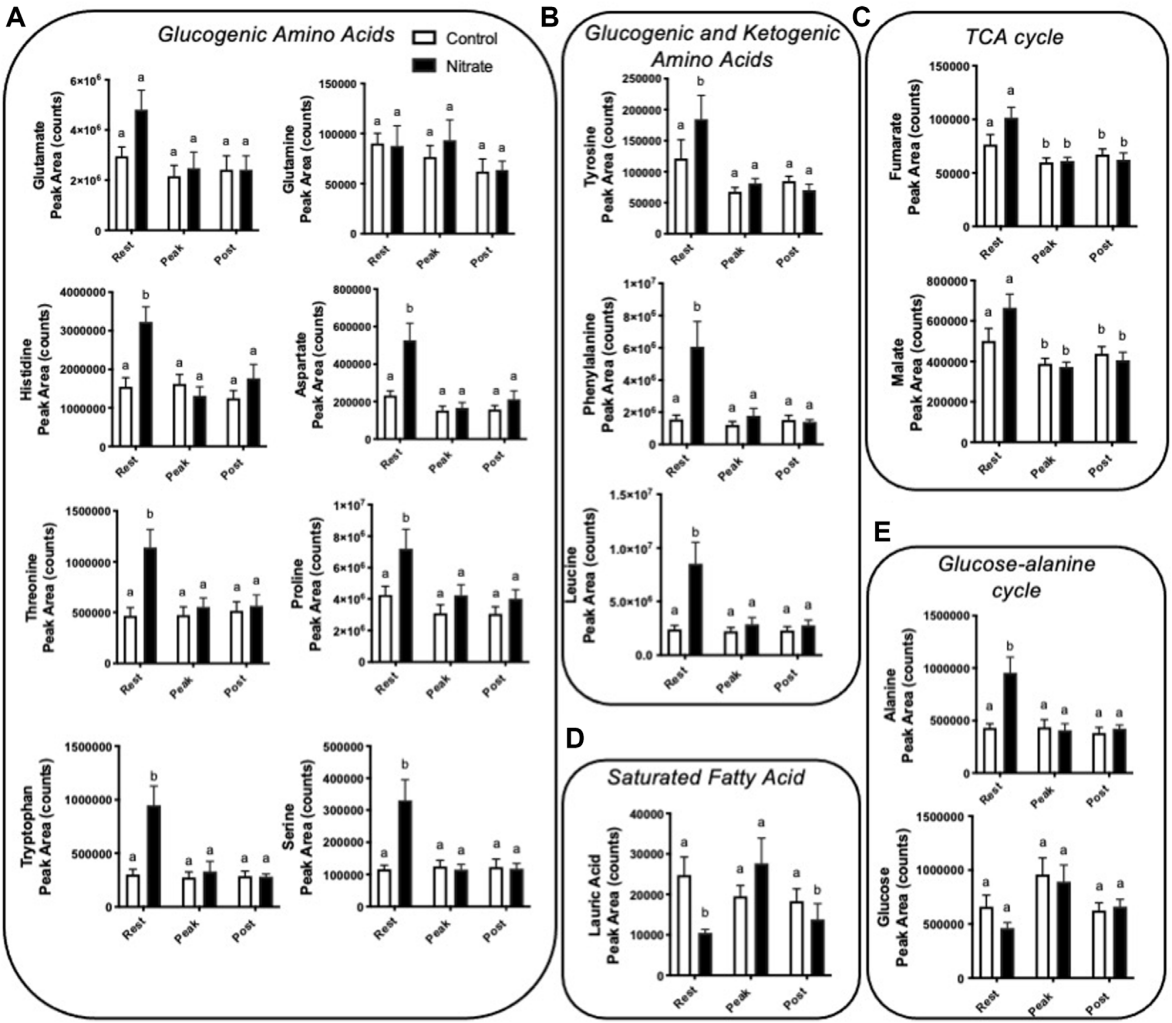
Nitrate exposure increased metabolites involved in amino acid metabolism, fatty acid metabolism, and the TCA cycle in liver. Labeled means without a common letter differ. (One-way ANOVA with Fisher’s post-hoc and Holm FDR-correction, *p* < 0.1 indicating significance, *n* = 7–9/group).

Amino acids contribute significantly to energy production in zebrafish. We observed several glucogenic and ketogenic amino acids that were significantly higher in rested nitrate liver compared to rested controls (Figure 3). Specifically, we observed an increase in glucogenic amino acids (arginine, aspartate, histidine, proline, alanine, serine, threonine, and tryptophan) by 1.7–3.1-fold (Figure 3A) and ketogenic amino acids (tyrosine, phenylalanine, and leucine) by 1.5–3.9-fold (Figure 3B) in rested nitrate-treated liver compared to control liver. With exercise, the abundance of these amino acids were reduced in nitrate-treated liver but no changes was observed in control liver with exercise. No change in glutamate or glutamine was observed.

### TCA cycle intermediates

We observed a greater abundance of the TCA cycle intermediates fumarate and malate in both rested nitrate and rested control liver compared to peak exercise and post exercise conditions (Figure 3C). Nitrate treatment did not significantly alter malate or fumarate with nitrate-treatment at any time point compared to control liver.

### Fatty acid metabolism

In nitrate-treated liver at rest, lauric acid was significantly reduced by 2.4-fold compared to rested controls (Figure 3D). At peak exercise, lauric acid was higher with nitrate treatment at peak-exercise compared to nitrate-treated liver at rest by 2.6-fold. No significant change in lauric acid, was observed in controls at any exercise condition. We did not see changes in palmitate, a primary fatty acid oxidized for energy metabolism (Supplementary Table S3).

### Glucose-alanine metabolism

Alanine, a prominent source of ATP production in the liver, was higher in nitrate-treated liver at rest by 2.2-fold compared to rested controls (Figure 3E). No significant difference in controls was observed for alanine at any exercise condition. Likewise, no significant change in glucose metabolite abundance was observed with nitrate treatment or control at any exercise condition.

### Dopamine synthesis pathway

**FIGURE 4:**
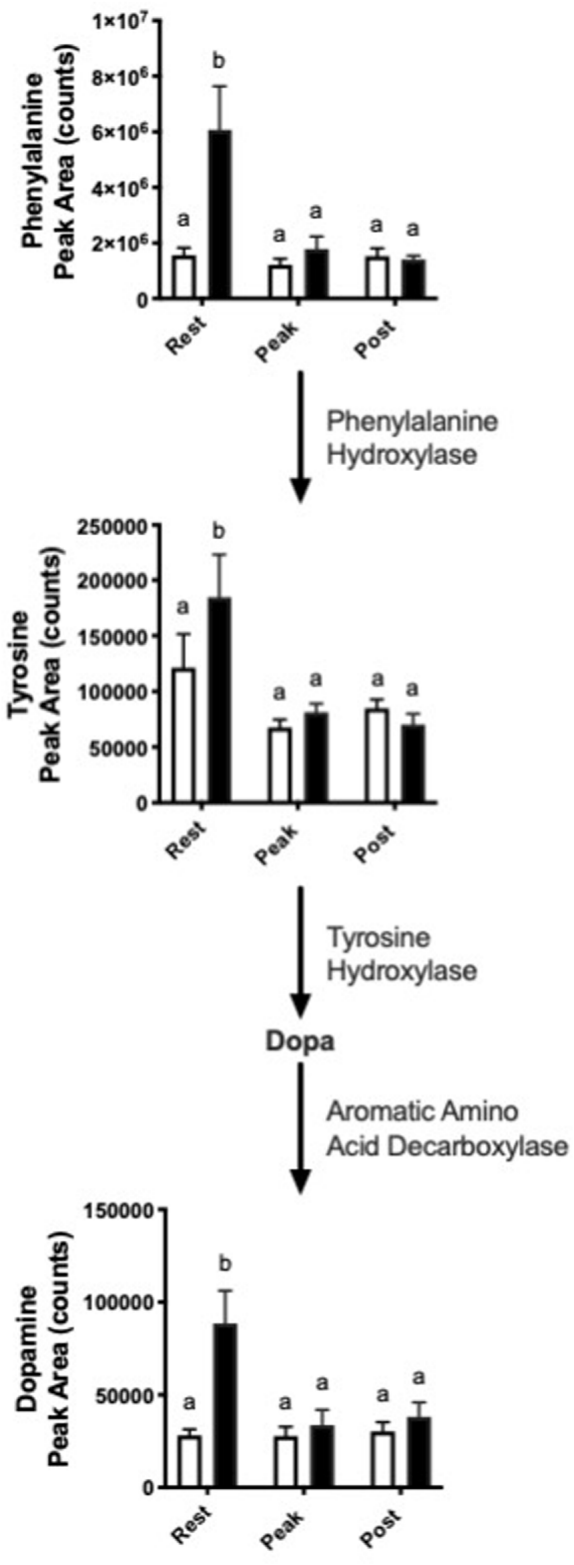
Nitrate exposure increased metabolites in the dopamine synthesis pathway. Labeled means without a common letter differ. (One-way ANOVA with Fisher’s post-hoc and Holm FDR-correction, *p* < 0.1 indicating significance, *n* = 7–9/group).

Interestingly, we observed increased abundance in several metabolites in the dopamine synthesis pathway with nitrate treatment at rest. (Figure 4). At rest, nitrate treatment increased phenylalanine, tyrosine, and dopamine by 1.5–3.9-fold, as compared to rested controls.

### Gene expression

**FIGURE 5:**
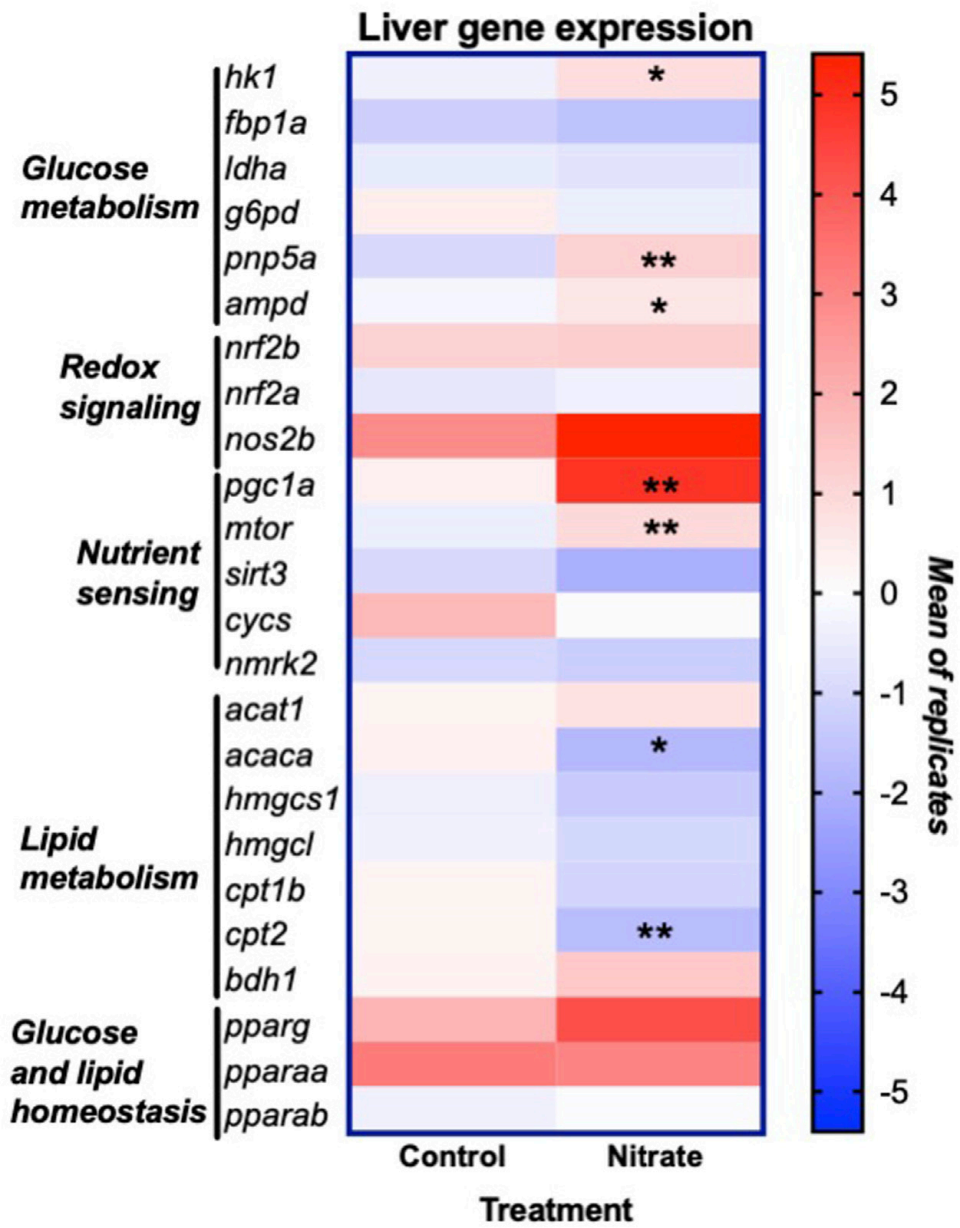
Expression of genes involved in liver energy metabolism and redox signaling resulting from nitrate treatment. The heat map was generated with Prism and color mapping represents the mean of each treatment group. Negative and positive values indicate downregulation and upregulation compared to control values, respectively. Asterisks indicate significance; *, *p* < 0.01; **, *p* < 0.001. (n = 6–8/group). *hk1*, hexokinase 1; *fbp1a*, fructose-1,6-bisphophatase a; *ldha*, lactate dehydrogenase a; *g6pd*, glucose-6-phosphate dehydrogenase*; pnp5a*, purine nucleoside phosphorylase 5a; *ampd1*, adenosine monophosphate 1; *nrf2a*, nuclear factor erythroid 2-related factor 2a; *nrf2b*, nuclear factor erythroid 2-related factor 2 b; *nos2b*, nitric oxide synthase 2b; *pgc1a*, peroxisome proliferator-activated receptor gamma coactivator 1-alpha; *mtor*, mammalian target of rapamycin; *sirt3*, sirtuin 3; *cycs*, cytochrome c oxidase; *nmrk2*, nicotinamide riboside kinase 2; *acat1*, acetyl-CoA acetyltransferase 1; *acaca*, acetyl-CoA carboxylase a; *hmgcs1,* 3-hydroxy-3-methylglutaryl-CoA synthase 1; *hmgcl,* 3-hydroxy-3-methylglutaryl-CoA lyase; *cpt1b*, carnitine palmitoyltransferase 1b; *cpt2*, carnitine palmitoyltransferase 2; *bdh1*, 3-hydroxybutyrate dehydrogenase; *pparg,* peroxisome proliferator activated receptor gamma; *pparaa*, peroxisome proliferator activated receptor alpha a isoform; *pparab,* peroxisome proliferator activated receptor alpha b

In order to glean possible underlying mechanisms that may contribute to nitrate-induced changes in liver metabolism, we examined the expression of genes that encode key enzymes and transcription factors involved in energy metabolism and redox signaling (Figure 5). This analysis was focused on the rested condition where most metabolic changes were noted. Nitrate treatment significantly upregulated hexokinase isoform 1 (*hk1*) (*p* = 0.0175), purine nucleoside phosphorylase isoform 5a (*pnp5a*) (*p* = 0.0022) and AMP deaminase isoform 1 (*ampd*) (*p* = 0.0289) compared to rested control liver. No significant difference in expression of fructose-1,6-bisphosphatase (*fbp1a*), lactate dehydrogenase isoform a (*ldha*) or glucose-6-phosphate dehydrogenase (*g6pd*) was observed between rested nitrate and rested control liver. Nitrate treatment significantly upregulated the nutrient sensors, namely peroxisome proliferator-activated receptor gamma coactivator 1-alpha (*pgc1a*) (*p* = 0.008) and mechanistic target of rapamycin (*mtor*) (*p* = 0.0023) compared to rested control liver. No significant differences were observed with cytochrome c oxidase (*cycs*)*,* nicotinamide riboside kinase 2 (*nmrk2*), or sirtuin 3 (*sirt3*)*, nrf2b, nrf2a*, or *nos2b* between rested nitrate and rested control liver. Nitrate treatment significantly downregulated acetyl-CoA carboxylase (*acaca*) (*p* = 0.0018) and carnitine palmitoyl transferase 2 (*cpt2*) in NRT muscle.

## Discussion

To our knowledge, this is the first study to report the effect of nitrate exposure and exercise on the metabolomic profile in whole liver. We measured the effect of nitrate and exercise to identify potential mechanisms by which nitrate treatment improves exercise performance. We observed a significant effect of nitrate treatment on glucogenic and ketogenic amino acid abundance, which was coincident with upregulation of *mtor* and *pgc1a* at rest, compared to rested controls. Glucogenic amino acids are metabolized to glucose, via gluconeogenesis in the liver, increasing glucose output to peripheral tissues (Jungas et al., 1992). In addition, ketogenic amino acids are ultimately degraded to CO_2_ in the TCA cycle and contribute to ATP production. As with our observations in whole zebrafish and zebrafish skeletal muscle (Axton et al., 2019; Keller et al., 2021), the effect of nitrate was most prominent at the rested condition and not at peak-exercise or post-exercise. A primary finding of our study is that 21 days of sub-chronic nitrate exposure significantly increased arginine bioavailability, sparing arginine and likely modulating endogenous NO metabolism. Similarly, to our previously published results in whole fish and zebrafish muscle, we observed a greater abundance of arginine in nitrate-treated liver at rest (Axton et al., 2019; Keller et al., 2021). Our data support that sub-chronic nitrate treatment may improve exercise performance, in part, by improving NO bioavailability, sparing arginine, and increasing indices of hepatic gluconeogenesis in the liver.

Nitrate treatment has been shown to increase nitrate liver nitrate storage and spare arginine locally (Gilliard et al., 2018). This suggests that nitrate treatment spares arginine by producing NO via the nitrate-nitrite-NO pathway. Indeed, nitrate has been shown to increase arginine by inhibiting arginase in the hypoxic rat heart, redirecting arginine from ornithine/citrulline production to NO/citrulline formation (Ashmore et al., 2014). We observed a greater abundance of ornithine at rest and no change in abundance of citrulline with nitrate exposure at rest compare to rested controls, possibly suggesting increased arginase activity. However, this effect of nitrate on arginase enzyme remains to be elucidated. Previous research in humans has shown that arginine supplementation improves exercise performance, similar to nitrate supplementation, by reducing the oxygen cost of exercise and extending time to exhaustion (Bailey et al., 2010). We previously showed that exogenous nitrate exposure in this model is converted to nitrite in blood, as occurs in humans, and that nitrate and nitrite treatment changed the abundance of metabolites related to endogenous NO production (Axton et al., 2019). NO and arginine supplementation have independently been shown to be reduce liver injury and enhance liver regeneration after liver resection, offering another possible benefit of nitrate for liver function (Cantre et al., 2008; Kurokawa et al., 2012). We cannot comment directly on the mechanisms of nitrate reduction to nitrite and NO directly in zebrafish liver but the sparing of arginine may support NO bioavailability and liver function in our model and could plausibly influence whole-organism metabolic efficiency. Future research should measure enzymatic activity of xanthine oxidoreductase in liver, which reduces nitrite to NO, to determine whether nitrate treatment alters hepatic NO metabolism (Lu et al., 2005; McNally et al., 2020).

Another key finding of this study was that nitrate treatment in liver had a greater abundance of glucogenic and ketogenic amino acids compared to rested controls. The primary role of the liver during exercise is to metabolize waste products of muscle metabolism and contribute to ATP production. During exercise, the liver is responsible for maintaining glucose homeostasis during exercise via glycogenolysis and gluconeogenesis. In zebrafish, the amino acids glutamine, glutamate, leucine and alanine contribute significantly to ATP production in the liver and skeletal muscle (Jia et al., 2017). Zebrafish also initiate gluconeogenesis during fasting similar to mammals and inhibition of the phosphoenolpyruvate carboxykinase gene (*pck1*) results in sustained hyperglycemia in zebrafish embryos (Jurczyk et al., 2011).

The increase in alanine and leucine with nitrate treatment were coincident with upregulation of *mtor* and *pgc1a,* two key nutrient sensing genes involved in glucose and lipid metabolism. The nitrate-mediated effects on the nutrient sensing genes, *mtor* and *pgc1a*, are of interest because they play a central role in regulation of cell growth, autophagy, and are involved in the training-mediated benefits of exercise (Wang et al., 2019). The zebrafish target of rapamycin (*mtor*) has 90% homology with mTOR, however its physiological role is less understood (Makky and Mayer, 2007). Arginine and leucine are two amino acids known to activate mTOR, both of which were elevated in nitrated-treated liver at rest compared to rested controls. In rainbow trout hepatocytes, leucine has been shown to activate the mTOR signaling pathway stimulating gluconeogenic pathways (Lansard et al., 2010).

The observed upregulation of *pgc1a* in zebrafish liver with nitrate treatment at rest suggests modulation of hepatic gluconeogenesis and lipid metabolism. PGC-1 α is a transcriptional coactivator that regulates hepatic gluconeogenesis and β -oxidation of fatty acids in the liver (Liang and Ward, 2006). Tissue culture studies have revealed that overexpression of PGC-1 α in primary hepatocytes drives the expression of gluconeogenic genes (Yoon et al., 2001; Liang and Ward, 2006). Upregulation of PGC-1 α is associated with increased hepatic glucose production in a fasted state (Liang and Ward, 2006). In liver, we observed an increase in alanine and leucine and a trend toward increased glutamine abundance with nitrate treatment at rest, further supporting a potential increase in gluconeogenesis and glutaminolysis for ATP production.

Unlike our previous results in whole zebrafish, we did not observe a change in glucose-6-phosphate with nitrate exposure or exercise. In nitrate-treated skeletal muscle at rest, we observed an increase in glucose and glucose-6-phosphate, likely from increased glucose uptake in muscle (Keller et al., 2021). It is important to note that we did not see changes in expression of genes central to gluconeogenesis including *fpb1a* and *g6pd*. Further research is needed on the effect of nitrate exposure on expression of phosphoenolpyruvate carboxykinase and post-translational modification, primary regulators of hepatic gluconeogenesis (Zhang et al., 2018). Furthermore, future research should aim to better understand the involvement of *mtor* and *pgc1a* on lipid regulation and glucose metabolism in zebrafish liver to determine the translational significance of this model to humans.

Initiation of fatty acid oxidation is tightly controlled by acetyl-CoA carboxylase (ACC) activity and is important in exercise because it diminishes ACC activity and increases fatty acid oxidation in muscle and liver (Carlson and Winder, 1999; Dean et al., 2000). More specifically, inhibition of ACC stimulates fatty acid oxidation and inhibits fatty acid synthesis. Long-term regulation of ACC is primarily regulated at the transcription level (Park and Kim, 1991). When ACC is downregulated, as we observed with nitrate treatment, fatty acid oxidation is favored, suggesting increased fatty acid oxidation in liver. This is coincident with a decreased abundance of lauric acid (C12), a saturated fatty acid, in nitrate-treated liver at rest compared to rested controls. Lauric acid was higher in nitrate-treated liver at peak-exercise compared to nitrate-treated liver at rest and subsequently reduced in nitrate-treated liver post exercise, likely indicating increased β -oxidation post-exercise. Studies using a high-fat diet in rodent models show that nitrate supplementation may be protective against high-fat diet-induced steatosis by preventing lipid accumulation (Cordero-Herrera et al., 2019). Further targeted lipidomic analyses are needed to quantify the effect of nitrate on the metabolism on a greater number of fatty acids in liver.

Notably, we observed a nitrate-dependent increase in abundance of dopamine and dopamine precursors in liver. The majority of dopamine is synthesized in the substantia nigra, tegmental area, and hypothalamus in of the brain (Best et al., 2009; Juarez Olguin et al., 2016; Xue et al., 2018). However, peripheral dopamine can be produced by the autonomic nervous system, gut epithelial cells, and immune cells such as dendritic cells, regulatory T cells, B cells and macrophages (Xue et al., 2018). About 50% of dopamine is produced in the gut by enteric neurons and intestinal epithelial cells, leading to increased dopamine concentration in the hepatic portal vein (Xue et al., 2018). Interestingly, a study in mice showed that stimulation of central dopamine D_2_ receptors increases plasma glucose levels by increasing hepatic glucose production through parasympathetic nerves (Ikeda et al., 2020). The increased abundance of dopamine in liver may be another mechanism by which nitrate exposure mediates hepatic glucose output. However, the significance of this effect of nitrate exposure on dopamine synthesis remains to be discovered.

Several challenges arose with our study including variability and potential sex differences in liver metabolism. Due to the small size of zebrafish liver, we pooled 2 livers for each sample and combined males and females, which may explain the variability observed within treatment groups, potentially confounding our results. Indeed, sex specific differences in oral nitrate-reducing bacteria in humans exists (Kapil et al., 2018). Our treatments cannot differentiate between the potential direct and indirect effects of nitrate through reduction to nitrite and NO. Methodological considerations include the static nature of the metabolomics data, as opposed to metabolic flux experiments, requiring us to make inferences based on relative abundance of metabolite concentrations at each time point. However, this model did allow us to gain insight into a wide array of metabolic pathways related to energy metabolism and aerobic exercise performance. These conditions give rise to opportunities for future experiments to explore the sex-specific effects of nitrate, rate of flux of nitrate-mediate fuel sources in liver during aerobic exercise, and the potential conservation of these mechanisms in humans.

In conclusion, this study aimed to use a metabolomics-driven, discovery-based approach to determine the performance enhancing effects of nitrate treatment on liver metabolism. Our unique study design allowed us to gain insight into the global metabolic effects of nitrate and exercise on whole liver in zebrafish. We have shown that nitrate exposure spares arginine in liver and alters arginine, amino acid and lipid metabolism which is coincident with upregulation of central nutrient sensing genes, *mtor* and *pgc1a.* These data suggest nitrate may improve aerobic exercise performance by increasing NO bioavailability and hepatic production of glucose, via gluconeogenesis. Our findings are significant because the nitrate-induced changes in gene expression and metabolism provide insight into the mechanisms by which nitrate can prevent liver steatosis in pre-clinical models (Cordero-Herrera et al., 2019). Furthermore, these data support our previous analysis that nitrate increases glycolytic capacity and may contribute to improved aerobic exercise performance in our model. Taken together with our results in whole zebrafish and zebrafish muscle, these data support a conclusion that nitrate induces multi-organ metabolic reprogramming to support improved exercise performance in zebrafish.

## Data Availability

The datasets presented in this study can be found in online repositories. The names of the repository/repositories and accession number(s) can be found in the article/Supplementary Material.
