# Supplementary material for: Nitrate exposure reprograms hepatic amino acid and nutrient sensing pathways prior to exercise: A metabolomic and transcriptomic investigation in zebrafish (Danio rerio)
Source: Front Mol Biosci. 2022 Jul 19;9:903130. doi: 10.3389/fmolb.2022.903130 (PMC9343839; doi:10.3389/fmolb.2022.903130)

Supplementary Figure 5. Partial least squares-discriminant analysis (PLS-DA) of A) control and nitrate liver samples at rest and B) control, nitrate, and quality control (QC) liver samples at rest.

A)

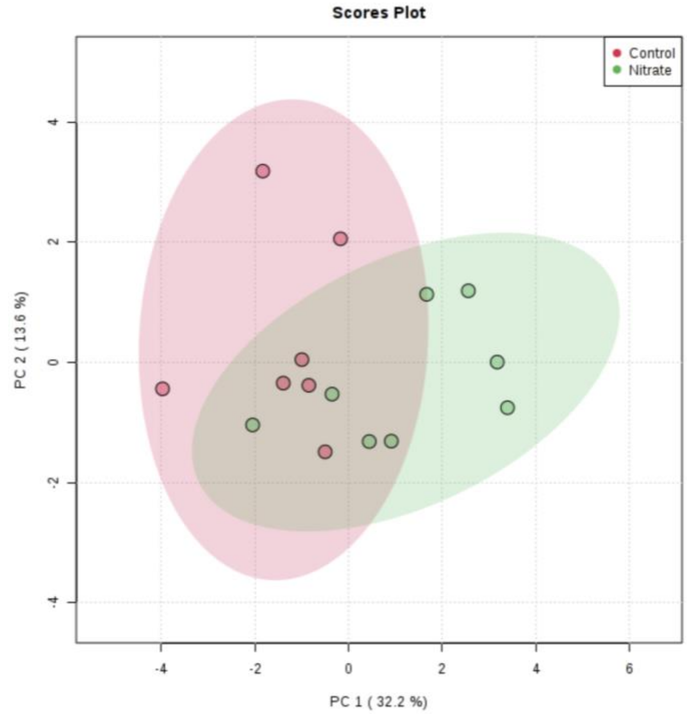

B)

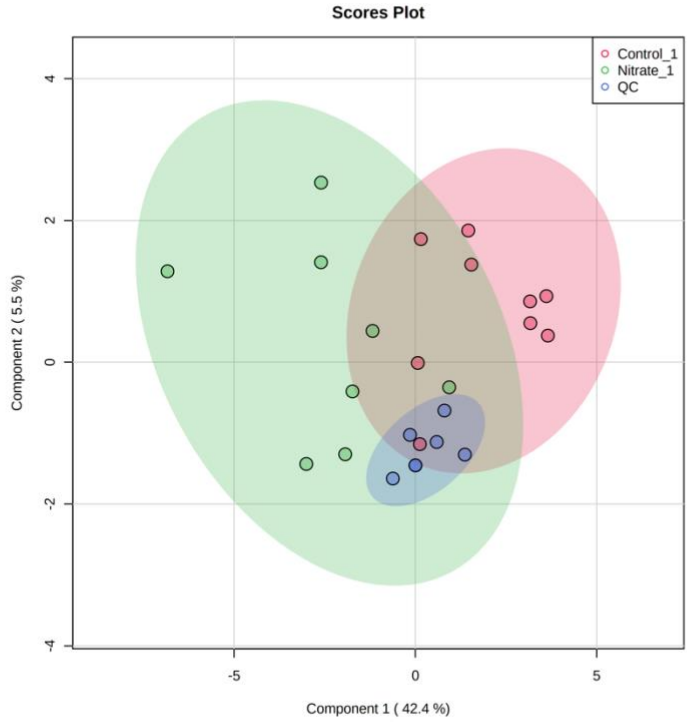

Supplement: Supplementary file 1 [file DataSheet2.PDF]
