# Supplementary material for: Nitrate exposure reprograms hepatic amino acid and nutrient sensing pathways prior to exercise: A metabolomic and transcriptomic investigation in zebrafish (Danio rerio)
Source: Front Mol Biosci. 2022 Jul 19;9:903130. doi: 10.3389/fmolb.2022.903130 (PMC9343839; doi:10.3389/fmolb.2022.903130)
Supplement: Supplementary file 3 [file Presentation1.PPTX]

## Slide 1
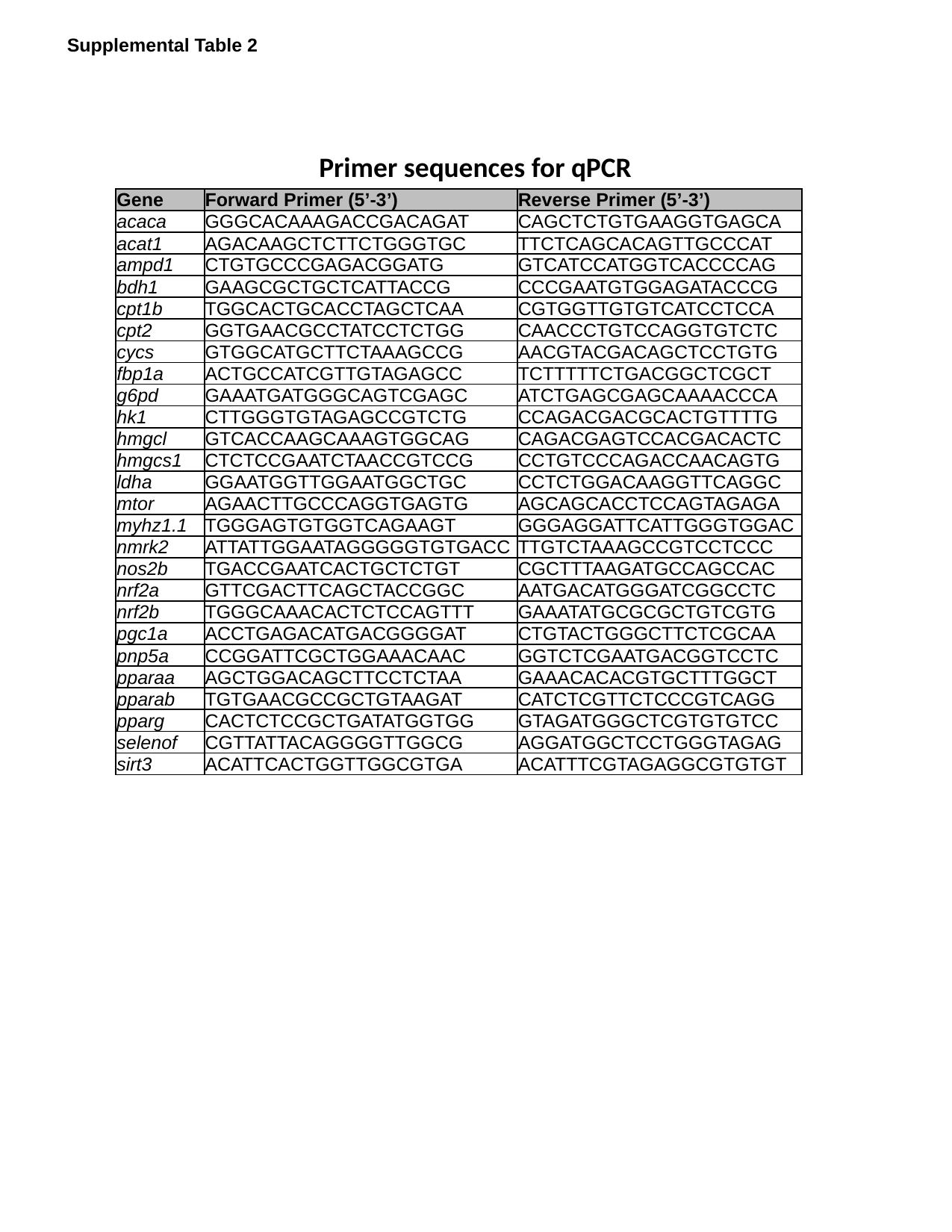

Supplemental Table 2
Primer sequences for qPCR
| Gene | Forward Primer (5’-3’) | Reverse Primer (5’-3’) |
| --- | --- | --- |
| acaca | GGGCACAAAGACCGACAGAT | CAGCTCTGTGAAGGTGAGCA |
| acat1 | AGACAAGCTCTTCTGGGTGC | TTCTCAGCACAGTTGCCCAT |
| ampd1 | CTGTGCCCGAGACGGATG | GTCATCCATGGTCACCCCAG |
| bdh1 | GAAGCGCTGCTCATTACCG | CCCGAATGTGGAGATACCCG |
| cpt1b | TGGCACTGCACCTAGCTCAA | CGTGGTTGTGTCATCCTCCA |
| cpt2 | GGTGAACGCCTATCCTCTGG | CAACCCTGTCCAGGTGTCTC |
| cycs | GTGGCATGCTTCTAAAGCCG | AACGTACGACAGCTCCTGTG |
| fbp1a | ACTGCCATCGTTGTAGAGCC | TCTTTTTCTGACGGCTCGCT |
| g6pd | GAAATGATGGGCAGTCGAGC | ATCTGAGCGAGCAAAACCCA |
| hk1 | CTTGGGTGTAGAGCCGTCTG | CCAGACGACGCACTGTTTTG |
| hmgcl | GTCACCAAGCAAAGTGGCAG | CAGACGAGTCCACGACACTC |
| hmgcs1 | CTCTCCGAATCTAACCGTCCG | CCTGTCCCAGACCAACAGTG |
| ldha | GGAATGGTTGGAATGGCTGC | CCTCTGGACAAGGTTCAGGC |
| mtor | AGAACTTGCCCAGGTGAGTG | AGCAGCACCTCCAGTAGAGA |
| myhz1.1 | TGGGAGTGTGGTCAGAAGT | GGGAGGATTCATTGGGTGGAC |
| nmrk2 | ATTATTGGAATAGGGGGTGTGACC | TTGTCTAAAGCCGTCCTCCC |
| nos2b | TGACCGAATCACTGCTCTGT | CGCTTTAAGATGCCAGCCAC |
| nrf2a | GTTCGACTTCAGCTACCGGC | AATGACATGGGATCGGCCTC |
| nrf2b | TGGGCAAACACTCTCCAGTTT | GAAATATGCGCGCTGTCGTG |
| pgc1a | ACCTGAGACATGACGGGGAT | CTGTACTGGGCTTCTCGCAA |
| pnp5a | CCGGATTCGCTGGAAACAAC | GGTCTCGAATGACGGTCCTC |
| pparaa | AGCTGGACAGCTTCCTCTAA | GAAACACACGTGCTTTGGCT |
| pparab | TGTGAACGCCGCTGTAAGAT | CATCTCGTTCTCCCGTCAGG |
| pparg | CACTCTCCGCTGATATGGTGG | GTAGATGGGCTCGTGTGTCC |
| selenof | CGTTATTACAGGGGTTGGCG | AGGATGGCTCCTGGGTAGAG |
| sirt3 | ACATTCACTGGTTGGCGTGA | ACATTTCGTAGAGGCGTGTGT |
