# Supplementary material for: Nitrate exposure reprograms hepatic amino acid and nutrient sensing pathways prior to exercise: A metabolomic and transcriptomic investigation in zebrafish (Danio rerio)
Source: Front Mol Biosci. 2022 Jul 19;9:903130. doi: 10.3389/fmolb.2022.903130 (PMC9343839; doi:10.3389/fmolb.2022.903130)
Supplement: Supplementary file 4 [file DataSheet1.PDF]

		Liver_1	Liver_2	Liver_3	Liver_4	Liver_6	Liver_8	Liver_9	Liver_10	Liver_11	Liver_12	Liver_13	Liver_14	Liver_16	Liver_17
Compound ID	Metabolite	Control	Control	Control	Control	Control	Control	Control	Nitrate	Nitrate	Nitrate	Nitrate	Nitrate	Nitrate	Nitrate
CPD01	Lactate	908000	441000	770000	823000	678000	1330000	689000	781000	647000	781000	913000	888000	691000	538000
CPD02	12-hydroxydodecanoic acid	41983	50301	72721	63565	33071	42449	60731	135000	52222	69387	33290	126000	39870	48585
CPD03	2-acetamido-2-deoxy-beta-d-glucosylamine	22805	51445	86597	39420	39284	71289	42821	202000	80399	83582	50103	186000	79717	114000
CPD04	2-amino-2-methyl-propanoate	228000	314000	104000	344000	161000	215000	301000	568000	138000	555000	421000	215000	123000	379000
CPD05	2-aminoethyl dihydrogen phosphate	3896.4	9397.5	6975.9	8247.6	4860.9	6868.3	9169.2	11498	10014	6030.6	6547.7	12667	4293.2	6896.2
CPD06	2-hydroxybutyric acid	63916	27263	28848	47621	30627	28626	27524	32099	29702	46227	66708	76966	58290	30212
CPD07	DMP	52568	45335	39821	59318	33160	129000	46750	79763	115000	151000	94541	132000	126000	55126
CPD08	4-aminobutanoic acid	228000	314000	104000	344000	161000	215000	301000	568000	138000	555000	421000	215000	123000	379000
CPD09	4-hydroxyproline	297000	602000	304000	300000	275000	775000	328000	781000	477000	969000	588000	620000	327000	413000
CPD10	5-aminolevulinic acid	297000	602000	304000	300000	275000	775000	328000	781000	477000	969000	588000	620000	327000	413000
CPD11	5-aminopentanoate	33400000	33100000	16200000	19500000	14000000	28700000	27700000	49900000	41600000	53300000	44600000	32100000	20800000	32600000
CPD12	5-oxoproline	57461	13663	63268	32872	59229	9130.3	18152	1534.1	19505	45817	39277	36470	26465	17919
CPD13	5'-methylthioadenosine	811000	997000	599000	106000	26369	269000	99221	291000	2220000	1550000	481000	793000	598000	60902
CPD14	A-D-galactose-1-phosphate	628000	388000	916000	706000	569000	216000	418000	633000	267000	393000	528000	495000	310000	650000
CPD15	Adenine	73196	32138	35312	21875	15966	17452	12749	14354	20832	33305	52945	117000	78532	15111
CPD16	Adenosine	640000	415000	138000	267000	154000	195000	182000	676000	524000	545000	479000	424000	440000	373000
CPD17	ADP	339000	396000	447000	100000	24966	84734	127000	533000	432000	527000	114000	118000	124000	117000
CPD18	ADP-ribose	72152	41988	36681	53025	17118	12281	28755	25536	36036	46991	41738	14616	20863	24939
CPD19	AMP	2960000	4860000	2720000	2570000	730000	3290000	3690000	5930000	5860000	6860000	2070000	7370000	3140000	3730000
CPD20	ADP-glucose	113000	355000	432000	51097	3024	83092	116000	519000	302000	527000	41460	116000	64032	40758
CPD21	Alanine	419000	396000	384000	432000	274000	630000	475000	944000	745000	1370000	605000	1570000	956000	498000
CPD22	Allothreonine	508000	782000	450000	260000	179000	409000	676000	975000	1080000	1500000	537000	1940000	1170000	786000
CPD23	Glucose-1-phosphate	64216	331000	204000	160000	97807	286000	239000	380000	210000	206000	205000	501000	123000	243000
CPD24	Aminoadipate	215000	79646	104000	103000	55502	84467	45459	313000	255000	307000	268000	276000	139000	104000
CPD25	Arginine	1340000	685000	738000	1190000	984000	832000	1890000	2680000	2370000	3000000	1580000	6460000	5030000	1650000
CPD26	Ascorbate	1642	11855	11845	2765.8	23877	97083	4000.6	23342	6637.7	7313.1	59779	8465.7	3977.1	20873
CPD27	Asparagine	135000	83119	89662	112000	66552	58651	67732	378000	149000	130000	127000	165000	108000	81717
CPD28	Aspartate	355000	265000	217000	254000	161000	179000	195000	939000	411000	644000	263000	659000	488000	287000
CPD29	Azelaic acid	64786	72963	79920	50797	95580	18290	42679	24717	24562	36018	55862	41087	27759	45947
CPD30	Beta-alanine	419000	396000	384000	432000	274000	630000	475000	944000	745000	1370000	605000	1570000	956000	498000
CPD31	NADP	19935	55225	61978	17262	10998	6206.6	5273.1	72713	30744	54635	5028.6	7511	5790.7	6002.9
CPD32	Betaine	33400000	33100000	16200000	19500000	14000000	28700000	27700000	49900000	41600000	53300000	44600000	32100000	20800000	33100000
CPD33	Caprylic acid	15451	24650	48439	1979.7	11007	14891	14199	291.9	13584	29019	2135.2	19175	2821.1	14914
CPD34	Carnitine	706000	1200000	288000	762000	466000	738000	1820000	1420000	944000	1340000	1040000	1180000	1530000	500000
CPD35	4-hydroxyproline	297000	602000	304000	300000	275000	775000	328000	781000	477000	969000	588000	620000	327000	413000
CPD36	Citrate	82534	404000	103000	26236	26577	146000	52196	109000	106000	88122	43663	45495	146000	29266
CPD37	Citrulline	204000	471000	325000	344000	189000	371000	566000	394000	576000	896000	386000	707000	352000	617000
CPD38	CMP	16086	107000	67041	44645	17914	47206	46861	55771	97191	96571	43636	77077	40169	53182
CPD39	Creatine	14500000	12200000	8840000	14600000	9260000	15900000	18800000	23400000	17100000	17500000	25400000	22300000	14900000	22100000
CPD40	Creatine phosphate dibasic tetrahydrate	116000	279000	264000	141000	46002	82844	131000	349000	276000	202000	223000	148000	113000	220000
CPD41	Crystathionine	887000	3580000	1720000	176000	134000	257000	184000	1810000	1060000	3150000	733000	4350000	2250000	229000
CPD42	Cytidine	72906	21050	24645	104000	155000	194000	153000	53918	38035	92340	52224	44962	62060	83356
CPD43	Cytidine 5'-diphosphocholine	134000	314000	194000	348000	123000	437000	514000	392000	401000	600000	380000	616000	319000	444000
CPD44	3-phosphoglyceric acid	4630.1	31138	37692	9511.6	6653.7	9751.8	7699.9	32151	41311	35431	8833.2	10289	10191	14768
CPD45	Galactosamine	34914	45811	57344	58256	37170	85832	59595	117000	35553	72477	56197	125000	35663	66599
CPD46	Fructose-6-phosphate	404000	417000	908000	705000	367000	218000	419000	631000	275000	392000	361000	372000	183000	649000
CPD47	Gluconolactone	8752.2	22275	4363.7	7106.8	9684	41715	14280	8397.9	25435	10209	11553	7476.2	8479.6	7301.8
CPD48	Mannosamine	34914	45811	57344	58256	37170	85832	59595	117000	35553	72477	56197	125000	35663	66599
CPD49	Ornithine	26983	14381	45662	12055	16652	15133	17754	36318	42359	104000	24359	94571	62603	15856
CPD50	Pantothenic acid	111000	269000	84725	167000	71591	193000	199000	165000	186000	254000	259000	288000	212000	316000
CPD51	Ribose-5-phosphate	66874	55000	65941	71163	63999	35629	86346	63798	99725	34673	54169	67872	48407	77199
CPD52	Deoxycarnitine	253000	860000	336000	576000	178000	609000	941000	1070000	844000	1250000	695000	1300000	935000	1170000
CPD53	Dopamine	42154	32892	26026	28766	14402	23480	29419	86860	92996	112000	36500	139000	135000	17156
CPD54	Elaidic acid	133000	128000	104000	184000	482000	388000	160000	97167	338000	277000	144000	58916	239000	286000
CPD55	Erucic acid	12569	4077.5	4623.1	8168.6	24921	6023	2738.6	13062	11584	31703	3666.6	1641.4	4718.3	11835
CPD56	Ethanolamine phosphate	23846	47982	21379	52814	23716	41109	50757	97462	72529	85168	53303	94891	52604	62564
CPD57	Ethyl-3-ureidopropionate	43284	99523	169000	66533	78591	108000	76915	434000	138000	149000	59942	252000	123000	171000
CPD58	Ethylmalonic acid	7563.2	3810	0	9219.3	8943.3	0	0	2658.6	0	6765.9	11302	0	7777.8	8459.4
CPD59	Flavin adenine dinucleotide	33849	239000	88698	22602	19953	10890	30019	50348	151000	88977	27329	8082.9	26964	24024
CPD60	Fumarate	137000	86613	53094	64069	60066	94072	82539	80451	129000	95247	79900	131000	124000	60424
CPD61	Galactarate	38767	57200	12037	14639	11853	41087	108000	8477.7	20110	19956	32694	53766	40559	13780
CPD62	Gluconic acid	213000	208000	112000	159000	173000	246000	167000	159000	307000	259000	171000	205000	233000	185000
CPD63	Glucosamine	34914	45811	57344	58256	37170	85832	59595	117000	35553	72477	56197	125000	35663	66599

CPD64	Glucose	459000	522000	621000	557000	455000	1260000	754000	517000	666000	376000	464000	578000	257000	380000
CPD65	Glucuronic acid	47450	59013	30457	61833	55503	34631	40873	45770	65486	51089	44465	44880	21240	66997
CPD66	Glutamic acid	2990000	3360000	2820000	4400000	1770000	3650000	1680000	8590000	4620000	5960000	3990000	5090000	2480000	2910000
CPD67	Glutamine	85724	137000	100000	67711	56844	78117	106000	113000	197000	71136	58385	76131	39185	58822
CPD68	Glutarate	1172.2	2651.3	2114.7	9132.4	9081	581.85	966.33	1529.9	295.85	6589.2	11648	442.19	8027.2	6825.5
CPD69	Glutathione	772000	3340000	1100000	2240000	1370000	9930000	9660000	1090000	2430000	1240000	14500000	14800000	13200000	15400000
CPD70	Glycerol 2-phosphate	432000	291000	557000	441000	499000	477000	568000	514000	702000	551000	426000	610000	278000	377000
CPD71	Glycine	20709	25278	12966	32603	19770	46653	43266	49794	38052	72858	27941	61388	43502	46481
CPD72	Guanine	119000	208000	21766	306000	62976	83779	77739	525000	78803	92753	179000	45630	77085	225000
CPD73	Guanosine	88044	48213	22770	119000	67338	2709.6	16565	80630	13183	15362	108000	13189	26100	78541
CPD74	GMP	196000	210000	187000	136000	70177	180000	254000	505000	289000	543000	220000	392000	279000	212000
CPD75	Glucose-6-phosphate	124000	331000	204000	160000	97807	286000	239000	380000	210000	447000	205000	501000	123000	243000
CPD76	Hippurate	2580000	2300000	1140000	2150000	1170000	4260000	2850000	2390000	3730000	8000000	4520000	3490000	5290000	3580000
CPD77	Histidine	2690000	880000	1940000	1310000	1680000	1210000	1160000	4230000	3200000	3400000	3720000	4330000	2170000	1540000
CPD78	Homoserine	508000	782000	450000	260000	179000	409000	676000	975000	1080000	1500000	537000	1940000	1170000	786000
CPD79	Hydroxyisobutyric acid	63916	27263	28848	47621	30627	28626	27524	17399	29702	46227	66708	76966	58290	30212
CPD80	Hypotaurine	12012	46711	24622	13466	9395	42621	46394	31664	67529	41857	18933	46498	38904	32743
CPD81	Hypoxanthine	1790000	1940000	707000	3150000	1890000	3000000	2490000	3540000	2790000	3790000	4280000	1480000	1960000	4020000
CPD82	Inosine	1190000	397000	265000	1010000	1270000	306000	501000	456000	400000	343000	1080000	268000	530000	999000
CPD83	IMP	438000	976000	486000	1050000	853000	973000	658000	2080000	398000	2190000	838000	940000	818000	1210000
CPD84	Inosine 5'-phosphate	451000	577000	548000	1230000	1760000	1230000	1120000	1150000	383000	1120000	1030000	592000	766000	1630000
CPD85	Isoleucine	3830000	3430000	2060000	1870000	1120000	2490000	2080000	6490000	8770000	9570000	3630000	18000000	11200000	2130000
CPD86	Kynurenine	76712	16001	10744	9614.4	12304	13609	14971	39652	92959	106000	36815	103000	12953	20185
CPD87	Aminocyclopropane-1-carboxylate	183000	418000	343000	273000	128000	215000	220000	681000	302000	791000	217000	547000	234000	306000
CPD88	Oleoyl-rac-glycerol	150000	66069	38934	710000	417000	365000	110000	621000	169000	618000	423000	297000	172000	290000
CPD89	Lauric acid	18792	39675	16080	14220	28404	41409	14928	9938.3	11058	10403	9622	12772	6646.8	13239
CPD90	Lauroylcarnitine A104	13856	4774.9	2981.8	32989	12561	17776	17242	20133	16656	37115	37560	25464	17228	41375
CPD91	Leucine	3830000	3430000	2060000	1870000	1120000	2490000	2080000	6490000	8770000	9570000	3630000	18000000	11200000	2130000
CPD92	Lysine	758000	334000	375000	596000	413000	476000	668000	1330000	1170000	1610000	1020000	2990000	1800000	667000
CPD93	Malata	900000	519000	362000	434000	398000	635000	554000	560000	767000	626000	491000	943000	806000	386000
CPD94	Maleic acid	132000	68563	52535	65120	62010	92285	80522	81585	86755	96330	80444	131000	125000	60393
CPD95	Menaquinone	2289.3	2091.3	2404.6	1293.2	2326.5	0	1863.8	340.2	0	2690.4	705.16	1191.3	996.44	1250.1
CPD96	Methylmalonate	240000	81850	66527	99698	77553	38157	67433	85827	140000	90288	159000	283000	226000	70626
CPD97	Myristic acid	25352	46163	47055	40361	45126	61534	35527	16023	44873	30826	35578	16634	16785	36618
CPD98	Acetylneuraminat	24111	20675	21200	40148	30195	25873	9614.7	27920	26863	22504	27214	16948	11240	20457
CPD99	Methyl-aspartic acid	279000	284000	276000	292000	201000	264000	148000	374000	389000	206000	116000	240000	102000	76981
CPD100	Methyl-glutarate	215000	79646	104000	103000	55502	84467	45459	313000	255000	307000	268000	276000	139000	104000
CPD101	Methyl-histidine	183000	255000	151000	260000	119000	261000	151000	481000	224000	434000	289000	321000	217000	272000
CPD102	NAD	440000	685000	488000	516000	425000	413000	351000	620000	319000	470000	415000	298000	332000	554000
CPD103	NE,NE,NE-Trimethyllysine	506000	1040000	222000	970000	1220000	449000	527000	736000	881000	1440000	1450000	752000	1160000	890000
CPD104	Nicotinamide	618000	769000	351000	1570000	661000	2160000	849000	755000	1260000	1800000	1410000	1040000	1080000	1390000
CPD105	Norvaline	33400000	33100000	16200000	19500000	14000000	28700000	27700000	49900000	41600000	53300000	44600000	32100000	20800000	33100000
CPD106	Acetyl-serine	279000	283000	272000	293000	200000	262000	148000	374000	386000	205000	205000	235000	72036	76981
CPD107	Phosphoserine	10563	16573	12016	17846	4941.8	11672	7865.1	32468	17176	18210	12370	16690	10607	10646
CPD108	Ophthalmic acid	50472	8108.8	6886.7	24616	25002	38898	3191.4	16044	0	6059.1	27866	6451.9	8972.6	10135
CPD109	Ornithine	26983	14381	45662	12055	16652	15133	17754	36318	42359	104000	24359	94571	62603	15856
CPD110	Palmitate	253000	688000	494000	280000	432000	571000	573000	275000	777000	535000	228000	192000	323000	681000
CPD111	Palmitoleic acid	20694	28950	17529	41080	86742	84589	34125	17546	53390	92454	34904	11751	23507	43025
CPD112	Petroselinic acid	133000	128000	104000	184000	482000	388000	160000	97167	338000	277000	144000	58916	239000	286000
CPD113	Phenylalanine	2470000	2610000	1130000	1390000	951000	971000	1370000	3350000	7480000	6020000	2180000	13200000	8760000	1520000
CPD114	Phosphocholine chloride	3310000	22400000	11900000	7080000	2980000	6820000	22000000	6390000	31600000	16200000	5730000	23700000	21800000	15000000
CPD115	Phosphocreatine	116000	279000	264000	141000	46002	82844	131000	349000	276000	202000	223000	148000	113000	220000
CPD116	Proline	3700000	4860000	4750000	3010000	1980000	6350000	5220000	7790000	8860000	13400000	6070000	6230000	3530000	4530000
CPD117	Putrescine	9076.1	5865.7	4517.8	23071	8904.7	46592	29007	9467.5	7931	20408	20738	57574	30859	27315
CPD118	Rac-glycerol-myristate	16071	5980	4485.5	63577	43133	37962	10025	67752	18042	70858	47246	29780	18033	26873
CPD119	Retinoate	292000	258000	173000	774000	484000	858000	285000	777000	327000	1140000	640000	549000	232000	395000
CPD120	Riboflavin	25266	17568	11224	24362	22246	35429	24844	52524	27385	66066	58012	37911	35238	33266
CPD121	Ribose	8102.6	13250	12389	9077.1	14877	18016	14556	9485.7	12650	8880.6	9030.4	13038	9500.4	14508
CPD122	5'-adenosyl-homocysteine	46957	41338	11840	18423	14472	17082	7897.5	15803	28308	37506	20468	49068	41180	22838
CPD123	Saccharic acid	38767	57200	12037	14639	11853	41087	108000	8477.7	20110	19956	32694	53766	40559	13780
CPD124	Sarcosine	419000	396000	384000	432000	274000	630000	475000	944000	745000	1370000	605000	1570000	956000	498000
CPD125	Serine	140000	164000	141000	87010	81734	91093	110000	284000	308000	365000	170000	659000	384000	150000
CPD126	Sodium benzoate	186000	224000	147000	37565	64791	11640	17107	36120	102000	42437	19836	8142	12139	14414
CPD127	Sodium gluconate	8752.2	22275	4363.7	7106.8	9684	41715	14280	8397.9	25435	10209	11553	7476.2	8479.6	7301.8
CPD128	Spermidine	347000	299000	381000	673000	494000	1060000	729000	250000	606000	674000	927000	1880000	1240000	1430000

CPD129	Sphinganine	69873	90119	192000	116000	78366	111000	103000	325000	145000	170000	67402	228000	140000	146000
CPD130	Sphingomyelin	118000	109000	59183	245000	155000	120000	93734	386000	210000	535000	169000	198000	135000	244000
CPD131	Stachyonse hydrate	72268	15588	58227	104000	84492	178000	39431	42809	18110	46751	61173	55726	15155	56493
CPD132	Suberic acid	17423	16388	32505	18597	31842	2621.1	11640	7527.5	5762.4	12147	7956	12087	6588.8	12958
CPD133	Succinate	240000	81850	66527	99698	77553	38157	67433	85827	140000	90288	159000	283000	226000	70626
CPD134	Sucrose	144000	25788	72472	286000	239000	731000	138000	174000	53189	190000	207000	233000	56103	121000
CPD135	Taurine	2540000	4970000	4600000	4150000	2310000	3400000	4720000	7250000	4800000	5870000	3990000	7790000	4640000	5500000
CPD136	Theophylline	226000	178000	93519	210000	103000	388000	237000	274000	330000	691000	414000	300000	447000	305000
CPD137	Threonine	508000	782000	450000	260000	179000	409000	676000	975000	1080000	1500000	537000	1940000	1170000	786000
CPD138	Tocopherol	243000	347000	85774	188000	65411	608000	374000	179000	488000	814000	435000	659000	337000	619000
CPD139	Trans-4-hydroxyproline	297000	602000	304000	300000	275000	775000	328000	781000	477000	969000	588000	620000	327000	413000
CPD140	Trigonelline	3000000	4290000	1080000	9440000	4780000	7850000	6490000	4290000	5550000	7630000	12900000	6130000	6620000	11600000
CPD141	Tryptophan	497000	447000	223000	267000	153000	210000	319000	648000	1190000	1210000	413000	1460000	1400000	305000
CPD142	Tyrosine	291000	102000	78124	124000	132000	66026	56165	203000	82757	195000	123000	358000	256000	74620
CPD143	Urate	170000	150000	76593	208000	103000	225000	296000	389000	341000	336000	215000	412000	296000	87293
CPD144	Uridine	79170	70200	16864	47961	48114	1944.9	39326	30765	37346	24915	44384	30334	55581	40050
CPD145	UDP	11804	40951	63798	27378	12168	10998	12845	46547	40008	103000	27874	34234	28515	41937
CPD146	UDP-acetylgalactosamine	18502	315000	276000	126000	61884	35162	162000	316000	313000	185000	71604	143000	93496	256000
CPD147	UDP-acetylglucosamine	18502	315000	276000	126000	61884	35162	162000	316000	313000	185000	71604	143000	93496	256000
CPD148	UDP-galactose	13676	212000	329000	51721	27963	44678	173000	140000	148000	123000	35646	50031	22533	110000
CPD149	UMP	127000	169000	164000	134000	149000	171000	427000	153000	230000	187000	164000	406000	318000	202000
CPD150	Valine	33400000	33100000	16200000	19500000	14000000	28700000	27700000	49900000	41600000	53300000	44600000	32100000	20800000	32600000
CPD151	Xanthine	62938	114000	30479	302000	119000	381000	263000	342000	203000	556000	408000	198000	241000	413000
CPD152	Aspartic acid	268.5613106	250.3561376	220.2187711	214.5296262	430.6595522	214.353831	171.1677188	238.2063557	179.1565341	498.5883441	166.3305193	171.6538411	565.9711942	481.215776
CPD153	Deoxycholic acid	1470.774477	667.9596063	894.4328531	922.0150397	1070.061993	959.0894618	1155.646871	173.2362093	690.7034903	781.9852508	311.7211618	1629.501938	650.8978849	1480.671277
CPD154	Phenylpyruvic acid	433.6657594	339.3821923	1180.994932	822.0747371	1455.905135	786.8038468	475.0601117	1043.916378	621.4780523	2479.765338	462.3805476	257.1863928	1421.037574	1289.891478
CPD155	Resveratrol 3-O-glucuronide	9.440968549	33.89203042	50.29584424	4.198577851	7.925216615	1.519134521	20.49930228	2.958861048	5.80569455	12.54371104	3.407367807	0.927032441	4.309634445	2.138366699
CPD156	Retinyl Beta-glucuronide	304.0538682	2.33391777	5.381504912	411.2656918	562.9301597	102.8453137	16.46002877	66.52168993	2.95982458	58.69302639	449.6346167	121.4489636	47.36349199	215.9537448
CPD157	Phenylacylamine	879.9834913	395.8777675	202.5164227	341.7114337	168.7413101	157.3063922	265.7455036	468.2540253	514.5134426	623.6171427	386.2951531	1178.079956	870.0518929	192.9989956
CPD158	Oleamide	1152.33116	1138.869808	452.8170479	951.6924282	1750.564821	780.506277	702.3863232	179.4757915	263.5561066	538.0192389	478.2738957	411.7639205	297.1954836	1012.049632
CPD159	Pipecolic acid	917.156332	453.1268107	492.3324559	531.6460594	604.7676605	692.3460103	871.0055569	1033.533384	1394.639202	1024.808028	1323.519817	2111.534262	1785.140901	574.8522127
CPD160	Linoleoyl Ethanolamide	573.0097949	252.0706296	18.43481953	125.3561842	324.5855966	0.665937884	24.72391139	355.99579	252.232772	1175.724694	657.7430985	1148.57081	887.8701961	271.7444221
CPD161	Alloisoleucine	10300.90162	10705.82773	7526.808972	2987.214881	3316.481235	6492.39177	5933.668606	9677.633765	17037.78866	12370.30292	7344.397995	23279.32207	18991.78173	4289.709537
CPD162	Kynurenine	174.4022746	61.22609311	0	0	21.41359482	29.85380272	4.428137109	29.71419174	272.0417908	63.79779639	63.20887543	119.1929799	36.30514038	17.86745368
CPD163	Argininosuccinic acid	133.8960599	2.076115142	50.55171033	8.900181972	28.39048807	15.53109708	126.7456835	222.119125	328.8177261	221.5341392	148.7158459	243.62713	399.2520304	10.89526304
CPD164	8,11,14-Eicosatetraynoic acid	158.0146798	33.9060286	17.64653619	117.4889201	1204.222377	3985.242739	172.7687359	64.7824676	442.7725207	614.3312763	653.4978097	72.33793173	355.4592168	165.7214322
CPD165	Hydroxyhexodocosahexaenoic acid	979.6610921	195.8972559	89.89189263	433.3163724	3521.431761	16798.49247	822.7136946	371.4969997	1829.755003	1737.064694	1666.598201	536.3537359	1123.409303	809.3078519
CPD166	Flavonol 3-O-D-galactoside	819.4833274	872.5789416	1237.183902	926.7695969	1259.404424	2154.413189	1500.489983	1202.514537	672.687364	699.8543791	1049.10641	853.5193116	924.3848498	1511.021927
CPD167	Monacolin J acid	47.47173075	108.3186545	58.67138618	120.235939	124.6163367	76.08623478	25.21522846	130.3015282	115.9042218	96.51861966	252.4407521	198.4843295	104.5971687	369.5094308
CPD168	Kryptogenin	194.6209561	19.3383894	6.423857954	4154.009306	1482.830634	3452.043684	447.7128037	138.5712603	23.81083116	181.7983673	1342.250784	182.7169423	387.8975358	106.5524813
CPD169	D-myo-Inositol 1,2-cyclic phosphate	40.40805107	5.164298774	16.89088538	37.12733969	66.36971201	182.2349406	165.6924394	29.38306511	15.62281125	58.75396873	22.58800874	19.43707126	24.8408674	71.81239407
CPD170	N-Hydroxy-L-valine	171.2465045	100.3294852	305.851339	42.25623384	84.90043746	60.45140355	105.8713143	211.546263	199.8885855	250.7469105	77.62467854	379.9181921	337.6879648	61.14223797
CPD171	Lipoic acid	68.50414468	14.95980179	6.742729523	27.94442011	38.79431352	35.91573694	59.91556045	59.00647047	87.00567973	72.1372277	80.47756911	145.2481625	164.0947368	42.94003056
CPD172	4-Sulfobenzoate	79.54811583	17.37282154	21.53119848	64.35404792	24.62709199	17.55794485	7.93078793	2.961159107	8.647237249	19.92671224	2.046374614	16.86563558	35.02250057	13.56042685
CPD173	4-Hydroxyphenylglyoxylate	60.12026128	67.8330518	13.04880484	28.52882941	34.30647046	10.76456481	7.697805811	9.938502197	12.73819765	8.62280699	5.649867876	10.26459893	20.49434862	5.309231468

Liver_19	Liver_20	Liver_21	Liver_22	Liver_23	Liver_24	Liver_25	Liver_26	Liver_27	Liver_28	Liver_29	Liver_30	Liver_31	Liver_32	Liver_33	Liver_34	Liver_35	Liver_36
Control	Control	Control	Control	Control	Control	Control	Control	Control	Nitrate	Nitrate	Nitrate	Nitrate	Nitrate	Nitrate	Nitrate	Nitrate	Nitrate
733000	709000	726000	1080000	1260000	910000	956000	863000	1010000	378000	798000	544000	636000	552000	811000	877000	392000	376000
165000	58112	47028	52846	25912	38168	25038	19419	60387	44511	42387	23115	42573	49332	115000	49911	19845	28319
208000	82473	65013	85956	29184	62141	85546	89760	113000	74861	72143	15299	45520	51585	175000	65523	84257	74136
684000	149000	195000	261000	153000	87885	177000	310000	265000	340000	286000	105000	343000	343000	666000	107000	230000	170000
3612.2	8098.4	4984.3	10016	10339	10637	8881	11154	0	1803.1	6960.8	4245.2	5479.8	6691.8	10033	13066	4874.9	13321
49545	34365	33686	43863	43485	29789	37807	41434	41142	38430	78344	42381	80075	54133	41122	1765.3	40629	46159
217000	129000	64362	49799	55129	24057	79227	49405	136000	50224	54839	23588	108000	101000	126000	55529	26413	10298
684000	149000	195000	261000	153000	87885	177000	310000	265000	340000	286000	105000	343000	343000	666000	107000	230000	170000
1440000	404000	437000	264000	184000	216000	296000	442000	154000	978000	927000	252000	409000	478000	648000	186000	120000	150000
1440000	404000	437000	264000	184000	216000	296000	442000	154000	978000	927000	252000	409000	478000	648000	186000	120000	150000
48400000	22600000	17900000	15700000	18200000	8810000	13700000	17200000	17900000	43100000	33800000	12500000	18500000	48000000	38100000	17900000	17000000	8460000
51449	14219	14960	60524	31577	19459	32030	21466	12391	7084.4	22310	41065	31816	22544	26583	8623.6	8692.6	27995
61955	208000	59916	57789	30058	60331	59502	68571	34290	308000	237000	23195	140000	421000	34190	71117	32244	26317
705000	300000	274000	813000	428000	643000	273000	497000	230000	282000	605000	494000	398000	618000	696000	357000	828000	879000
14838	5319.1	11950	8979.4	9760.4	40483	20700	9793.3	11180	14548	20182	8852.6	14590	58197	24893	11244	15718	28340
284000	269000	122000	196000	63144	63851	190000	241000	86229	260000	236000	41778	140000	93471	328000	40605	412000	237000
364000	843000	424000	48461	98761	130000	87897	239000	65119	321000	620000	316000	119000	111000	81701	87106	110000	90607
44146	27221	40533	19771	317000	21024	31573	46451	27234	52012	73136	570000	35757	29190	30876	11422	9057.1	23214
6450000	6310000	3080000	1610000	1720000	1640000	1980000	2580000	1440000	3120000	3130000	1290000	1630000	4040000	4010000	2210000	2260000	1110000
364000	781000	424000	17509	35386	130000	35972	199000	22976	243000	617000	295000	30411	41627	57071	48065	96895	27008
892000	577000	409000	322000	190000	194000	532000	325000	489000	406000	527000	191000	520000	504000	744000	372000	220000	194000
485000	732000	653000	245000	423000	167000	900000	254000	398000	413000	838000	257000	635000	952000	634000	729000	285000	232000
915000	335000	257000	142000	171000	133000	293000	177000	298000	303000	319000	126000	185000	343000	362000	238000	208000	125000
219000	112000	85244	48588	40911	40154	52835	63475	129000	89592	92270	27157	100000	53790	126000	55529	35446	35479
978000	877000	885000	820000	788000	651000	1930000	857000	1220000	1220000	835000	531000	3340000	2540000	1940000	1740000	1200000	890000
0	13617	17037	25628	19695	94714	3494.3	5703.7	141000	6801.4	13026	26357	12249	27913	3200.8	13045	20046	113000
152000	52724	57682	69572	28258	41005	71339	65142	64841	101000	62268	24163	248000	47678	165000	42559	69707	40124
266000	205000	129000	119000	63644	81443	243000	122000	139000	207000	119000	70526	218000	167000	358000	127000	97793	132000
74392	36655	49755	108000	48880	56285	53170	55283	71862	46476	10562	49817	52986	34177	91977	39901	42438	49745
892000	577000	409000	322000	190000	194000	532000	325000	489000	406000	527000	191000	520000	504000	744000	372000	220000	194000
71930	45220	31038	12753	3846.7	22022	5624.1	58355	0	60538	45853	72389	9359	6338.4	11923	0	4206.3	23954
48400000	22600000	17900000	15700000	18200000	8810000	24600000	17200000	17900000	43100000	33800000	12500000	18500000	48000000	38100000	17900000	17000000	8460000
59160	22514	14696	23317	29055	21187	16334	18010	2318.9	26415	3250.8	307.53	1415.3	2460.7	39200	6967.6	2373.1	44086
876000	1150000	633000	510000	460000	495000	908000	489000	545000	579000	1030000	420000	1040000	456000	1050000	328000	576000	304000
1440000	404000	437000	264000	184000	216000	296000	442000	154000	978000	927000	252000	409000	478000	648000	186000	120000	150000
73005	106000	109000	31666	34138	21427	46372	52864	34538	117000	118000	53193	28222	71820	20073	16423	14043	10179
690000	681000	309000	241000	316000	127000	430000	374000	259000	354000	916000	149000	453000	957000	580000	512000	373000	212000
167000	172000	71356	44526	21412	9839.2	45481	60889	53522	83529	127000	38171	65750	77561	50717	33130	39031	24209
32900000	19600000	14000000	10800000	7350000	7260000	14100000	12300000	14500000	15200000	24400000	5230000	26700000	16200000	27500000	9270000	10900000	6490000
488000	385000	275000	74797	78052	52746	88048	127000	139000	269000	662000	104000	332000	298000	214000	139000	122000	95745
536000	6070000	3350000	196000	430000	533000	416000	179000	288000	368000	2790000	550000	262000	4370000	437000	699000	881000	293000
53366	19260	19404	64396	55705	66768	58565	47821	51118	137000	40174	37842	77901	51916	70587	42683	50853	81033
491000	1080000	411000	163000	228000	167000	424000	223000	379000	173000	645000	177000	286000	672000	594000	347000	332000	150000
20726	51431	40225	8132.4	9084.4	14131	16069	52288	13065	29174	32754	73970	9174.8	10591	5922.6	14879	4266.6	0
311000	117000	46119	64849	45655	45085	74724	58819	78279	114000	58743	38816	68743	73128	268000	80423	60607	47562
708000	313000	276000	811000	428000	649000	267000	633000	227000	379000	610000	494000	204000	373000	694000	275000	829000	707000
12120	59487	7940.2	9182.7	12199	12461	9802.3	14259	48642	53240	8820	18523	31333	8544.3	11794	54896	9279.5	21684
311000	117000	46119	64849	45655	45085	74724	58819	78279	114000	58743	38816	68743	73128	268000	80423	60607	47562
74795	14065	13101	21364	17516	8221	31100	10239	25038	10849	15892	17029	11948	62583	31364	18846	20323	10061
144000	90568	89768	62892	75555	42309	114000	38173	118000	110000	97226	41160	154000	144000	172000	42277	57242	34462
57528	66526	52853	87846	67041	74822	75381	80794	44479	43005	34126	86251	29883	55632	71703	46120	46645	80276
964000	1570000	881000	165000	765000	300000	877000	443000	559000	468000	1850000	274000	1180000	990000	756000	557000	574000	277000
45130	49694	38339	19430	15157	12631	27243	6720.3	35630	25555	33593	9745.7	93268	42669	29724	33364	24960	9930.9
149000	153000	93896	348000	139000	104000	311000	477000	437000	1460000	157000	281000	884000	116000	255000	156000	286000	381000
12161	4748.8	4962.3	16287	6155.5	9569.3	6223.3	21952	12621	82859	9665.6	17044	19826	2458.4	17422	0	11186	24297
71569	87058	38092	44416	30781	26020	48025	46154	50982	34858	55161	19620	68834	71830	112000	70379	27990	39235
346000	115000	84750	155000	52902	61489	119000	117000	150000	96071	111000	40429	83021	84163	270000	92372	124000	108000
1413	4295.1	0	3333.6	6288.1	4897	3300.9	7454.7	5101.5	0	12617	7118.6	13329	69597	6083.8	10814	0	0
116000	177000	77123	13334	22646	26909	18509	114000	39078	135000	104000	108000	37524	23826	21840	14527	23812	20648
43765	76362	65560	42350	55432	56726	74061	66867	57861	57957	64400	50898	82053	69084	46810	56138	59737	63267
23297	6921.8	32710	6192.8	37102	8671.7	56681	14976	25353	13236	31007	28448	25821	75183	30458	45664	27363	6037.2
291000	78292	215000	150000	170000	275000	233000	135000	165000	225000	170000	188000	111000	179000	190000	190000	104000	133000
311000	117000	46119	64849	45655	45085	74724	58819	78279	114000	58743	38816	68743	73128	268000	80423	60607	47562

458000	1640000	417000	918000	892000	804000	1010000	740000	1750000	1530000	495000	689000	1060000	576000	874000	1720000	421000	677000
47246	37248	45382	72080	64688	67190	46895	72026	61507	64365	43725	32059	36391	38977	26676	53033	37533	77183
5170000	2390000	1590000	1910000	817000	904000	1540000	2850000	2240000	2860000	1720000	806000	4280000	1850000	6750000	1340000	1650000	1050000
39345	126000	36370	92843	45786	70579	65255	90381	124000	105000	22736	65617	146000	21609	92752	204000	50156	133000
2720	2153.9	3388.9	3590.1	4085.9	4543.7	3007.1	7573.8	3202.3	545.93	12611	6364.8	13647	71307	4254.8	1237.4	1814.4	1396.9
16800000	2260000	5140000	3550000	4950000	391000	9630000	6400000	10700000	689000	5400000	306000	11800000	15600000	8240000	5860000	9720000	1330000
697000	339000	433000	518000	349000	455000	498000	544000	430000	349000	362000	328000	303000	303000	552000	273000	230000	517000
91968	54628	38866	30406	20400	19816	53286	27719	45112	22951	38237	14417	57777	48082	69187	35872	20228	13988
448000	83574	62251	200000	59689	35423	28843	254000	157000	229000	111000	66817	412000	189000	250000	21575	104000	58200
49395	15720	14230	73229	33306	59434	36404	85939	23007	27305	40874	31742	68358	56459	66728	14428	49084	73137
752000	577000	302000	118000	107000	90493	174000	286000	233000	343000	320000	133000	160000	298000	259000	172000	143000	97296
915000	335000	445000	142000	171000	133000	293000	177000	298000	303000	538000	171000	185000	709000	362000	238000	208000	125000
6290000	4410000	2460000	1320000	1680000	1150000	2590000	1480000	3620000	3190000	3650000	1090000	2480000	5070000	5300000	2600000	1220000	775000
2640000	1710000	2790000	1340000	756000	626000	1650000	1540000	1570000	1680000	1850000	648000	990000	1160000	2850000	1140000	804000	677000
485000	732000	653000	245000	423000	167000	900000	254000	398000	413000	838000	257000	635000	952000	634000	729000	285000	232000
49545	28811	33686	43863	43485	29789	37807	41434	41142	24012	78344	42381	80075	54133	40858	32404	40629	19181
45415	137000	29874	16910	25203	29663	46076	15803	22818	15815	85760	9686.6	20493	49087	22772	31520	21050	8640.1
4850000	2300000	2380000	2240000	1480000	926000	1600000	2580000	3060000	2880000	2460000	842000	4370000	4630000	4330000	1130000	1410000	1070000
495000	173000	595000	898000	663000	707000	705000	826000	613000	354000	592000	689000	1060000	853000	782000	294000	506000	1080000
2020000	1270000	1370000	548000	453000	412000	717000	768000	1010000	1090000	1600000	608000	807000	1040000	1750000	1290000	443000	332000
960000	713000	1580000	788000	1080000	1160000	678000	885000	784000	687000	1340000	1180000	725000	1110000	1080000	1610000	1070000	1120000
3660000	3770000	2480000	1270000	1400000	774000	2580000	1460000	2640000	2380000	3750000	920000	6690000	4550000	2930000	2320000	1550000	808000
28055	37390	52170	22098	19993	12546	36034	27044	335000	48068	33195	22835	15684	46564	29951	33598	13326	6574.5
389000	269000	215000	163000	135000	99465	240000	209000	186000	179000	225000	91598	369000	418000	491000	216000	160000	118000
307000	106000	73523	372000	41812	78913	195000	478000	306000	2500000	301000	67785	1170000	80648	580000	55578	296000	490000
13104	27369	9398.4	23619	19695	19056	10807	34022	18858	24706	12029	30814	23601	10705	29869	31050	13547	72742
34409	36781	7388.2	22073	6374.2	5665.8	13027	25658	16028	42005	23615	2685	71610	17501	22863	7376	23010	3729.8
3660000	3770000	2480000	1270000	1400000	774000	2580000	1460000	2640000	2380000	3750000	920000	6690000	4550000	2930000	2320000	1550000	808000
864000	454000	712000	466000	481000	282000	920000	497000	754000	547000	593000	282000	1450000	1440000	1100000	714000	566000	365000
269000	430000	430000	294000	379000	399000	526000	441000	333000	332000	457000	320000	484000	450000	290000	319000	351000	354000
41698	76129	67822	43512	56199	57821	75198	67021	58411	51923	65240	50235	66727	70167	44361	49763	57004	64681
5452.2	1180.8	2684.9	1400	3195.4	4119.4	3354	2415.4	1967	588.29	622.72	3039.6	962.78	956.46	6513.1	0	3643.5	0
61540	71423	62929	71112	50934	78902	98106	71424	71552	61535	91392	62699	154000	94449	72292	46865	64615	102000
19502	26330	15946	62533	27586	39110	49734	90099	78054	158000	12090	42136	77523	15379	37650	45540	26478	69978
31933	13591	35006	23280	11788	17731	14226	25216	22415	35493	26135	21787	7451.9	6651.9	16926	13182	17755	30317
118000	204000	106000	194000	118000	169000	125000	276000	242000	262000	79576	122000	228000	68457	290000	212000	130000	220000
219000	112000	85244	48588	40911	40154	52835	63475	129000	89592	92270	27157	100000	53790	126000	55529	35446	35479
649000	254000	172000	180000	85410	78606	115000	147000	160000	329000	259000	73536	316000	198000	426000	113000	127000	81639
860000	614000	416000	484000	198000	403000	461000	673000	479000	519000	544000	279000	506000	306000	550000	349000	430000	736000
580000	240000	262000	428000	189000	513000	408000	374000	1390000	1280000	408000	549000	1810000	1760000	1210000	219000	465000	150000
1810000	854000	774000	830000	819000	246000	910000	1120000	2390000	1320000	1460000	605000	1790000	1420000	1930000	655000	281000	397000
48400000	22600000	17900000	15700000	18200000	8810000	24600000	17200000	17900000	43100000	33800000	12500000	18500000	48000000	38100000	17900000	17000000	8460000
68802	205000	106000	194000	118000	104000	50779	275000	238000	261000	79184	76123	275000	68286	286000	210000	128000	219000
13703	11529	6412.6	7276.6	3698.9	4938	6650.8	10080	6531.3	9542.5	9089.6	2608.7	13342	7860.2	23860	3686.8	5852.3	3418.4
32572	0	7475.6	14472	0	5923.2	2012.8	8003.8	0	13405	1934.2	1916.6	7501.7	2147.2	7018.4	0	2458.9	7507.8
74795	14065	13101	21364	17516	8221	31100	10239	25038	10849	15892	17029	11948	62583	31364	18846	20323	10061
514000	707000	469000	680000	485000	642000	687000	839000	1120000	1370000	391000	626000	877000	328000	724000	735000	613000	742000
24167	22748	22326	50324	19162	45408	67379	121000	86791	376000	41154	68095	310000	19580	56250	36556	48696	141000
149000	153000	93896	348000	139000	104000	311000	477000	437000	1460000	157000	281000	884000	116000	255000	156000	286000	381000
1510000	2630000	1270000	567000	853000	553000	1510000	737000	1270000	1800000	2330000	560000	4890000	2610000	1430000	949000	983000	449000
5640000	26200000	13500000	2940000	13700000	4740000	12500000	1350000	18600000	2660000	17800000	3550000	8510000	26900000	2780000	9420000	8930000	1450000
488000	385000	275000	74797	78052	52746	88048	127000	139000	269000	662000	104000	332000	298000	214000	139000	122000	95745
5600000	3160000	3440000	2520000	2050000	1540000	5770000	1520000	2250000	6060000	3870000	1890000	4990000	5880000	7490000	4060000	2000000	1950000
10190	9284.7	5043	10148	12233	14287	28525	9345.1	14791	7053.7	11343	1897	23223	60281	22144	27844	11366	9162.4
31784	6883.6	7930.5	29133	2842.8	11549	21356	64617	30525	387000	27453	7676.2	165000	7407.1	37924	7433.7	27780	56466
352000	218000	223000	467000	84687	103000	329000	605000	613000	2190000	463000	80256	1240000	223000	643000	197000	271000	335000
34219	15749	15414	16971	12430	8612.1	34905	37948	30487	27854	21625	12686	46251	28279	43500	10803	31604	13614
11073	25334	13341	20776	21918	18643	21954	18278	31122	24884	12438	10700	26501	17117	15033	31878	17755	22790
16157	28238	27694	21550	18135	29722	17480	14080	11238	7819.5	32754	24959	37131	36098	21065	9041.8	28368	5178.5
23297	6921.8	32710	6192.8	37102	8671.7	56681	14976	25353	13236	31007	28448	25821	75183	30458	45664	27363	6037.2
892000	577000	409000	322000	190000	194000	532000	325000	489000	406000	527000	191000	520000	504000	744000	372000	220000	194000
226000	171000	148000	75556	81731	65362	167000	66706	124000	81570	144000	53717	168000	155000	172000	133000	73111	57719
55610	115000	121000	68740	48464	67738	15795	24384	15700	95319	31438	254000	24568	36942	47926	19160	10599	14585
12120	59487	7940.2	9182.7	12199	12461	9802.3	14259	48642	53240	8820	18523	31333	8544.3	11794	54896	9279.5	21684
957000	686000	118000	645000	533000	336000	700000	274000	771000	301000	359000	71601	1360000	2060000	1430000	587000	429000	635000

299000	145000	110000	157000	55793	71497	64795	38323	123000	97384	95047	59291	74100	109000	224000	132000	45006	67629
545000	279000	156000	243000	51108	33781	94305	345000	252000	223000	132000	30425	262000	172000	538000	36818	112000	82185
112000	17836	25740	217000	41613	86890	38969	131000	89096	77697	18396	22817	61276	15527	121000	40713	81164	80967
28179	4201.8	9961.6	32876	11608	13162	15753	20160	13265	16362	3137.7	15453	9047.9	17277	31930	12532	15557	9606.8
61540	71423	62929	71112	50934	78902	98106	71424	71552	61535	91392	62699	154000	94449	72292	46865	64615	102000
424000	36273	72846	246000	73918	277000	159000	263000	400000	322000	91504	176000	227000	36058	410000	167000	148000	419000
9950000	6950000	4820000	3540000	3190000	2280000	4620000	3000000	4400000	3480000	5610000	2070000	5390000	6940000	7590000	4740000	3750000	2790000
526000	400000	216000	116000	145000	105000	240000	126000	362000	300000	353000	114000	223000	434000	496000	216000	116000	66976
485000	732000	653000	245000	423000	167000	900000	254000	398000	413000	838000	257000	635000	952000	634000	729000	285000	232000
1490000	644000	381000	108000	29276	14295	388000	731000	772000	220000	270000	19932	316000	362000	1470000	216000	626000	29282
1440000	404000	437000	264000	184000	216000	296000	442000	154000	978000	927000	252000	409000	478000	648000	186000	120000	150000
2790000	3770000	3420000	2490000	1980000	2260000	3890000	3450000	5770000	2930000	3200000	1260000	2570000	3480000	3690000	2880000	1820000	1370000
278000	478000	242000	137000	157000	95784	385000	167000	526000	241000	395000	94124	975000	531000	304000	169000	196000	74725
42609	45495	71069	94368	66846	94224	78203	77645	38390	66768	79800	112000	115000	52275	65457	61645	76903	102000
491000	439000	298000	161000	234000	6047.7	354000	176000	287000	153000	337000	32011	406000	530000	292000	159000	200000	12468
17095	16140	22757	43040	28743	32016	55635	36774	33488	14730	32558	24429	84741	22116	26676	0	36341	85672
174000	120000	58210	13533	11635	20284	9501.3	41451	36421	33786	51977	38746	20630	6291.4	36915	41219	25199	15476
240000	355000	323000	52236	102000	133000	112000	108000	151000	144000	434000	217000	200000	110000	50437	108000	127000	91758
240000	355000	323000	52236	102000	133000	112000	108000	151000	144000	434000	217000	200000	110000	50437	108000	127000	91758
262000	305000	358000	55345	148000	80707	91217	112000	87720	110000	493000	266000	35681	89775	51770	95841	85278	144000
207000	241000	372000	242000	380000	246000	497000	241000	143000	110000	320000	198000	87263	319000	129000	215000	261000	253000
48400000	22600000	17900000	15700000	18200000	8810000	13700000	17200000	17900000	43100000	33800000	12500000	18500000	48000000	38100000	17900000	17000000	8460000
740000	323000	303000	128000	317000	70600	314000	284000	340000	279000	287000	53970	364000	714000	560000	154000	190000	73173
218.6970132	77.43219293	252.7801073	243.1350212	137.2780495	486.7658525	334.3434219	134.3742701	399.5152231	253.3187481	242.7502526	512.5464182	429.2368874	274.6878665	168.8206063	236.4346355	650.7526104	927.8413034
953.900176	724.4582518	971.2993369	1060.109269	888.2024212	1042.017268	662.6529738	944.8448291	7173.776859	1260.140991	3737.523333	921.1706321	1063.711181	916.9232825	1201.600006	1043.76165	783.4372904	944.8913623
911.6744717	219.2920455	786.4404078	549.182629	243.5940373	3059.726291	2602.324994	377.0554429	968.3681618	1314.209308	1143.601874	1593.894392	971.6143136	1370.00277	693.0902719	788.2493987	4956.253881	5357.621341
28.67235151	12.7536138	30.59131246	101.4486249	166.3580884	123.4030364	172.6936819	480.5689504	35.25965685	2.876668829	3.79989624	20.7626102	101.4237926	29.80266333	15.29238004	6.316075139	8.474285037	1.070571929
93.93333083	166.5582558	385.0307557	481.8290216	4110.458987	434.2977081	1442.787708	131.6648741	168.5172874	114.9815626	448.7002211	1025.393425	108.9996809	2220.444534	47.09116553	193.6444767	126.7163574	112.6727472
201.6057755	165.8940461	233.373818	217.2657644	213.7143962	247.8371045	222.130402	264.6002386	243.9839586	280.4725875	102.0340561	308.0871309	461.2721177	235.8314638	110.529898	178.626448	174.7252659	211.6296346
659.9797425	371.8327479	463.5073679	824.8731224	24.66615941	146.2405922	438.9043326	404.4252015	972.2961289	402.6340703	205.8571905	37.21267463	537.5281139	373.6185124	580.6270394	279.0735905	536.9518744	86.05721363
482.5089137	621.0669479	1104.940226	513.0695357	1395.988955	839.5589714	1389.110088	625.3170645	858.8733757	638.2476755	889.757736	1183.679129	1017.593764	1789.669212	798.0667818	1658.734409	945.1754641	761.1404343
22.2212918	19.62430855	286.1523945	12.13050416	0	0	0.619814923	0	2.437970065	449.3822249	1798.767808	10.74490117	44.44914574	3.683391989	63.98212934	143.0964626	25.78008642	10.21352939
4151.622989	7523.720179	6715.338543	3046.941594	7526.447958	4210.035378	7832.561372	2960.762016	5683.648312	4963.53714	9141.109531	7233.130845	7838.651907	10942.24839	4072.780596	8465.203293	4964.869178	3028.304882
7.392262505	57.33946787	167.1240097	35.47091706	37.3284343	14.8689965	89.03797687	7.786010068	789.6641103	48.87690624	55.80289057	35.58304384	10.88987963	106.6442661	14.34240771	80.4490718	27.12478752	1.345224487
13.99407412	7.898657472	66.00826878	35.78089915	18.39169352	13.88602435	121.2264101	51.20397482	39.69938193	37.25501768	13.2330814	38.50714349	15.55168994	60.7190753	62.74598766	138.7429461	11.07563271	19.42514076
19.99658178	18.33809364	410.6860841	292.1531406	21.44492064	425.6182545	175.0517747	572.2928223	135.730537	1020.720105	1411.914379	440.3733431	260.6745138	180.7854184	96.76061616	221.1779121	155.0180391	372.6779887
206.810917	103.3120171	1249.405879	954.4711459	191.7609425	1402.381268	666.2955541	2008.735734	447.2951259	2724.009049	5382.062679	719.694528	1032.950145	565.9204676	361.5704308	1255.147498	480.0797753	2059.674348
3818.27472	2932.342744	1613.656361	1619.658405	2353.414462	2686.420998	2315.025469	1159.717694	2032.932899	1319.353026	1582.047131	2861.911917	895.0056991	3380.097574	1889.418217	3252.813886	1901.841833	2199.294683
50.52695412	2.828802955	12.67648083	52.3954462	6.387283789	141.1935861	15.3720704	74.62468728	21.95961087	53.82205731	4.586105734	176.5863308	144.7435563	2.357053203	32.05106221	0	341.4971345	565.2901819
44.90776503	80.42184263	29.76441313	297.540559	176.462027	390.2341084	165.7687629	4646.47337	651.0429293	142.2967598	27.42726322	41.98413646	3388.197008	631.8043823	172.2443918	155.0560179	5795.483708	918.6101354
296.8865414	120.9330583	203.9837278	36.27033405	254.9118905	139.2599467	222.8216898	102.4918541	315.706679	99.77640375	227.5671825	238.927956	28.89566007	217.2026419	146.6214763	161.1995624	91.04496039	93.28439277
179.126412	60.49927899	61.71659818	62.9971347	154.3931287	54.28358109	145.3819406	41.92414561	72.20156993	24.97133921	78.84932592	164.1382373	82.43429851	198.4125011	108.2694129	277.8681245	95.3108231	85.96087794
25.25327688	21.09847406	70.81880162	38.18888963	60.9335864	23.00466554	81.75688433	25.22962348	50.16884038	33.16705966	45.18063723	52.28730639	80.79891661	95.69579667	38.08968365	71.10454906	50.80748774	29.09363346
0.377573206	2.542886281	14.09276217	10.77538677	97.16248287	8.37201805	8.349320194	11.36633694	0	56.15026395	14.07410443	18.95684847	1.172661252	11.66750372	4.380533469	0.419203232	2.891483315	31.05673267
5.276507266	5.789482433	26.46153383	9.368958651	69.97183282	13.1186049	7.255652006	16.85422177	0.258119956	23.86590032	13.93204655	15.7781912	24.65859948	17.22000578	9.377289134	9.322163736	6.371815362	8.918317705

Liver_37	Liver_38	Liver_39	Liver_40	Liver_41	Liver_42	Liver_43	Liver_44	Liver_45	Liver_46	Liver_47	Liver_48	Liver_49	Liver_50	Liver_51	Liver_52	Liver_53	Liver_54
Control	Control	Control	Control	Control	Control	Control	Control	Control	Nitrate	Nitrate	Nitrate	Nitrate	Nitrate	Nitrate	Nitrate	Nitrate	Nitrate
468000	781000	850000	801000	609000	1070000	880000	518000	638000	428000	710000	618000	610000	736000	862000	1050000	724000	688000
8544.9	125000	61019	61654	59834	30350	28611	42438	47539	55446	123000	41927	161000	48256	141000	49516	19092	37022
55604	171000	77954	68578	119000	34249	30644	45496	39962	79755	192000	73119	183000	34497	189000	99911	30998	59719
445000	125000	82953	693000	136000	432000	92620	188000	337000	540000	298000	235000	499000	392000	571000	236000	321000	329000
4162.1	10036	4904.2	5327.8	7978.5	6058.8	9265.4	11536	5135.2	3435	17115	5382	4616.5	4569.6	12864	9708.6	4145.4	6838.4
42897	34206	35997	56106	37350	55631	75278	75674	37113	52875	51270	35039	30023	61782	39241	55044	114000	47012
32630	104000	25349	124000	73421	69451	51132	8096.4	71618	19136	38870	24489	39692	79183	48546	110000	74129	37005
445000	125000	82953	693000	136000	432000	92620	188000	337000	540000	298000	235000	499000	392000	571000	236000	321000	329000
483000	1030000	344000	531000	254000	210000	221000	81150	228000	1020000	558000	428000	528000	296000	393000	277000	159000	91629
483000	1030000	344000	531000	254000	210000	221000	81150	228000	1020000	558000	428000	528000	296000	393000	277000	159000	91629
28200000	33700000	5590000	36500000	34200000	17300000	19300000	20000000	29300000	25100000	26800000	7780000	17400000	35300000	39000000	40300000	22700000	36900000
17147	38934	12822	61277	39210	50972	30220	30223	30645	22366	40230	21342	38887	44268	70948	16513	26051	31528
57952	224000	79473	290000	78337	75303	78025	44551	248000	162000	129000	54505	57206	434000	29544	92287	121000	98698
789000	549000	714000	487000	628000	675000	393000	539000	635000	984000	878000	761000	950000	177000	624000	284000	284000	319000
12027	21362	19592	48036	24870	28103	40328	22226	33296	12376	13743	9020.6	20765	81186	26219	17846	35259	24546
302000	475000	38417	865000	319000	208000	354000	139000	343000	238000	105000	85038	467000	203000	688000	85568	409000	269000
382000	411000	161000	222000	200000	69762	145000	105000	97559	551000	446000	382000	240000	219000	72465	85378	155000	183000
63604	32280	23300	36966	35355	44431	33953	28591	30209	51206	24270	20528	121000	63840	16162	12342	13766	21519
2330000	8080000	1190000	4620000	6600000	1930000	2030000	1780000	2910000	5950000	3170000	1400000	2950000	5890000	1970000	3540000	4540000	4320000
382000	402000	51162	140000	130000	51823	74157	40871	63847	308000	152000	382000	203000	219000	37019	20236	137000	87096
377000	511000	197000	647000	396000	502000	284000	131000	392000	397000	419000	315000	563000	384000	597000	487000	367000	277000
244000	1020000	320000	469000	821000	282000	403000	392000	684000	339000	659000	373000	445000	1350000	577000	706000	232000	393000
181000	421000	241000	216000	366000	138000	134000	158000	235000	288000	549000	176000	228000	198000	260000	470000	235000	126000
98830	135000	41498	126000	132000	64000	67139	36627	74304	80025	59844	52850	111000	73880	95830	63656	75850	125000
527000	2330000	607000	1420000	2960000	885000	889000	849000	1910000	978000	1200000	648000	2240000	1900000	1450000	1860000	1130000	671000
4433.9	1598.6	11688	5807.5	53550	4121.7	4920.1	23450	2328.5	3231.8	119000	13705	0	231000	0	6214.6	11865	185000
90222	102000	18271	128000	58217	87749	28925	41261	81671	124000	77695	44491	228000	17944	163000	38431	54017	69295
206000	231000	55428	204000	209000	189000	90915	88221	146000	263000	204000	165000	496000	142000	334000	111000	90823	108000
29946	66270	47914	66286	63045	39019	56373	14188	35236	36109	20100	35365	49936	47040	145000	47494	34850	32393
377000	511000	197000	647000	396000	502000	284000	131000	392000	397000	419000	315000	563000	384000	597000	487000	367000	277000
30677	30972	16343	13908	7141.5	10930	5602.2	7345	9981.3	37268	40830	46035	25705	10781	15981	4633.2	4215.8	11187
28200000	33700000	5590000	36500000	34200000	17300000	19300000	20000000	29300000	25600000	26800000	16100000	17400000	47100000	39000000	40300000	22700000	36900000
5853.3	13256	3877.5	56883	18015	5755.9	3272.8	3872.9	15773	6983.2	16590	15059	29451	9949.8	35501	1558.9	5406.5	5784
1120000	2150000	317000	2090000	2360000	1250000	1400000	1010000	1330000	1330000	541000	753000	840000	2080000	1100000	1450000	503000	721000
483000	1030000	344000	531000	254000	210000	221000	81150	228000	1020000	558000	428000	528000	296000	393000	277000	159000	91629
239000	52571	48397	94738	18765	45019	38361	59905	72226	53043	107000	65645	27508	279000	40921	25846	20822	182000
273000	742000	195000	649000	718000	294000	377000	373000	431000	435000	612000	397000	372000	878000	371000	664000	499000	381000
68680	220000	39419	59746	89762	29365	33583	34829	55126	86779	129000	75380	72916	60992	50851	48043	64002	51633
10600000	33700000	5720000	22100000	27100000	11500000	12100000	9450000	18200000	17900000	14000000	9270000	20700000	27200000	17000000	14900000	9480000	12900000
172000	436000	86497	174000	285000	68537	114000	174000	230000	377000	308000	165000	75803	243000	111000	291000	56206	141000
430000	2710000	1520000	282000	597000	219000	776000	239000	381000	305000	4570000	1610000	320000	2200000	431000	536000	887000	63503
33293	45217	34844	48228	78090	77220	81358	48868	46271	23296	42780	31753	78905	52374	73658	60799	38598	53056
427000	837000	163000	495000	626000	320000	358000	193000	410000	673000	520000	325000	346000	691000	348000	557000	724000	603000
25040	25843	10755	13251	8344.5	7081.8	14896	7689.8	8971.9	23458	20715	29060	5453.4	8190	6883.4	6219.8	11214	9807.2
42693	136000	126000	92457	82575	55808	36612	35413	89348	96721	153000	46948	152000	73511	149000	94504	74221	35523
596000	544000	716000	380000	624000	434000	263000	532000	369000	983000	870000	761000	943000	172000	614000	396000	284000	330000
6633	3185.8	11021	20806	6501	11920	13139	2013.5	8153.6	6977.6	6966	4265.4	6173.5	9987.6	14959	15088	19289	8895.5
42693	136000	126000	92457	82575	55808	36612	35413	89348	96721	153000	46948	152000	73511	149000	94504	74221	35523
8951.8	50210	16451	23080	39256	12729	13061	7964.8	18628	13717	61349	7615.4	47297	33181	48957	27279	13694	7985.5
98439	173000	38697	143000	171000	109000	116000	91428	89807	104000	115000	59508	78662	169000	104000	134000	68038	115000
53585	60276	69279	58822	64665	69300	70243	66932	70609	67648	106000	77478	102000	41912	62032	68495	75317	70638
459000	1590000	366000	825000	1360000	322000	966000	855000	932000	480000	1330000	589000	440000	901000	467000	1380000	369000	632000
18850	48897	11484	45775	49872	31854	15325	18378	31976	26773	31256	15534	59732	37565	26833	36999	89820	18800
338000	122000	143000	272000	383000	246000	291000	85639	277000	227000	28665	66667	334000	298000	340000	167000	501000	242000
31347	3524.7	7216.8	9654.6	8806.5	16295	3111.3	1398.4	10084	13804	1635	4368.2	11963	6127.8	10078	4130.3	10857	5214.6
31316	97617	17959	50206	73094	18117	43507	44968	37506	27631	95660	33186	47741	35008	94946	54937	22935	26989
71114	222000	143000	106000	131000	50459	44660	70167	75731	122000	285000	97563	235000	59155	279000	138000	48662	82148
68248	5425	0	8079	38295	4751.3	75326	34486	10672	3069.9	5139	2226.7	2731.8	3082	5728.9	7398.6	4435.2	37637
138000	79042	53481	39319	20640	21529	25821	10282	12544	126000	29580	90280	44501	39619	56585	17873	22953	29725
63308	55702	35018	67721	66930	86394	66168	83752	79870	69888	49650	52244	43205	80094	39512	56747	69069	100000
11675	50532	8970.2	43703	51630	29020	7159.2	95849	50421	14941	41010	8384.2	16332	74046	18981	43705	50390	37904
173000	130000	196000	139000	104000	210000	197000	103000	144000	165000	122000	197000	134000	147000	111000	298000	126000	142000
42693	136000	126000	92457	82575	55808	36612	35413	89348	96721	153000	46948	152000	73511	149000	94504	74221	35523

274000	473000	989000	798000	431000	731000	668000	678000	589000	435000	584000	470000	694000	576000	989000	946000	773000	491000
50108	38162	40870	44521	38055	62317	55727	36812	43257	42885	52980	75702	41021	48594	65338	75412	41507	53921
3730000	2080000	705000	6070000	2100000	2850000	1020000	1020000	2160000	3340000	1790000	1570000	5130000	1730000	5080000	1490000	3190000	3530000
42677	18386	35662	138000	51255	87252	56981	92351	37676	59808	55395	42646	91935	31307	109000	36736	58191	87796
68255	4628.8	0	7870.9	37905	4878.7	75345	10271	10408	2134.7	2707.5	2202.2	2026.9	2822.4	5498.6	6660.7	3142.7	38741
5850000	6260000	777000	1230000	13000000	3850000	420000	5110000	6740000	1820000	3160000	2020000	4090000	13100000	3330000	10600000	11800000	11200000
361000	357000	423000	492000	356000	768000	326000	295000	444000	382000	389000	414000	574000	254000	472000	422000	471000	246000
20181	64177	21977	38682	56557	30507	32287	17479	28042	23071	40671	28868	45243	15416	51924	47651	23495	18852
335000	238000	15330	266000	140000	138000	78502	157000	215000	68293	178000	42265	234000	104000	180000	50476	88316	188000
114000	51758	14446	68640	65025	74382	62672	148000	79723	41860	55815	51696	73939	41483	78319	19853	49781	97612
266000	648000	120000	237000	502000	140000	125000	102000	248000	477000	310000	237000	287000	232000	159000	173000	203000	211000
181000	421000	241000	216000	366000	138000	202000	158000	235000	288000	549000	176000	228000	442000	260000	470000	235000	126000
2750000	3070000	1340000	5020000	3750000	2030000	1510000	637000	2690000	3800000	1920000	1810000	2930000	4630000	2050000	4750000	1730000	1280000
1080000	2190000	685000	2050000	1770000	1110000	675000	804000	870000	1040000	1870000	1250000	2450000	1680000	4300000	1600000	757000	959000
244000	1020000	320000	469000	821000	282000	403000	392000	684000	339000	659000	373000	445000	1350000	577000	706000	232000	393000
42897	34206	35997	56106	37350	55631	75278	75674	37113	52875	51270	35039	30023	61782	39241	30083	114000	47012
13686	79356	25338	19095	117000	18393	60824	22549	57991	19075	59252	25254	19235	88946	21208	40283	55546	34083
3190000	2830000	799000	4530000	2110000	773000	1770000	1410000	2830000	2190000	1770000	1120000	2950000	2050000	2770000	2790000	1430000	1440000
750000	406000	354000	865000	500000	492000	991000	1100000	710000	433000	220000	293000	811000	551000	817000	631000	665000	606000
1390000	2210000	560000	679000	1850000	793000	544000	724000	1470000	1450000	1090000	1330000	1370000	1420000	1120000	1090000	513000	859000
1380000	1240000	1190000	662000	1490000	1070000	1070000	1480000	1560000	955000	998000	1600000	772000	1420000	914000	1150000	751000	1220000
1780000	3490000	705000	3470000	3600000	1830000	1780000	1480000	2660000	2290000	2170000	1310000	2930000	3510000	1830000	3060000	6200000	1780000
18076	18710	6759	34481	14129	34501	23414	3183.5	22901	15164	15293	4507	38110	33844	15315	39580	8530.8	12145
238000	317000	107000	424000	297000	203000	128000	133000	236000	248000	304000	147000	368000	388000	383000	229000	230000	275000
516000	443000	43146	284000	567000	474000	105000	58935	306000	311000	102000	76475	831000	196000	268000	55058	379000	106000
11454	18880	13863	41057	18765	16579	15419	19115	10074	6731.2	10670	8443.4	19190	16678	41924	7908.1	9954	3136.3
28420	19892	4827.4	39666	29360	21252	10019	16831	17460	26319	3547.4	6036.3	25697	21562	18506	7221.3	11455	17231
1780000	3490000	705000	3470000	3600000	1830000	1780000	1480000	2660000	2290000	2170000	1310000	2930000	3510000	1830000	3060000	6200000	1780000
316000	1090000	337000	920000	1130000	534000	385000	445000	961000	517000	648000	349000	1250000	1240000	750000	1030000	361000	475000
403000	345000	231000	446000	421000	582000	479000	507000	526000	466000	320000	348000	292000	507000	237000	400000	487000	597000
64736	55878	35799	67104	67755	87054	66472	83263	80213	71456	49575	51127	40081	80598	46585	48761	69668	99544
414.07	1416.3	1352.8	6371.1	1072.8	496.98	2682.8	0	613.97	2979.8	0	0	2576.8	1695.1	11314	349.67	1637	519.34
63114	60193	54610	111000	76215	148000	141000	111000	118000	101000	56670	61013	66421	178000	94091	104000	133000	152000
39620	14956	25370	34481	27960	44979	27731	18472	37696	22719	16320	15229	48654	28510	58373	18084	37118	30323
42125	20023	8423.3	14322	7129.5	27845	43159	11138	26548	37520	7863	15488	17666	35171	18675	22876	24360	38088
92322	80000	86688	354000	96450	136000	54226	147000	118000	151000	127000	85026	241000	86772	299000	90341	261000	221000
98830	135000	41498	126000	132000	64000	67139	36627	74304	80025	59844	52850	111000	73880	95830	63656	75850	125000
218000	303000	97464	367000	204000	171000	104000	74755	177000	324000	261000	149000	353000	175000	250000	168000	121000	96629
686000	407000	411000	618000	338000	758000	296000	598000	407000	768000	419000	596000	382000	425000	485000	343000	278000	433000
681000	1190000	634000	1130000	1010000	829000	418000	203000	673000	2460000	276000	178000	1180000	556000	800000	346000	487000	973000
891000	1080000	298000	1390000	1020000	1040000	685000	314000	856000	769000	444000	422000	1920000	2350000	723000	1330000	582000	709000
28200000	33700000	5590000	36500000	34200000	17300000	19300000	20000000	29300000	25600000	26800000	16100000	17400000	47100000	39000000	40300000	22700000	36900000
91908	77085	83576	352000	95550	243000	54169	147000	118000	148000	126000	123000	241000	83370	297000	41554	261000	289000
15681	5985.8	2961.5	27511	11766	10138	3784.6	5196.5	7361.5	11279	9203.5	5941.9	18744	5741.3	16780	4770.5	11764	15697
20321	3224.9	0	20210	1602	23522	2695.2	9011.7	7707.7	14386	0	4087	10685	2959.7	4184.2	0	4650.5	2105
8951.8	50210	16451	23080	39256	12729	13061	7964.8	18628	13717	61349	7615.4	47297	33181	48957	27279	13694	7985.5
563000	243000	816000	558000	557000	343000	524000	338000	671000	460000	237000	315000	551000	419000	1320000	665000	958000	608000
77280	19405	27466	50692	61905	60588	50854	13872	51009	39855	7185	8939.2	60211	42042	72032	27350	93996	38971
338000	122000	143000	272000	383000	246000	291000	85639	277000	227000	28665	66667	334000	298000	340000	167000	501000	242000
980000	2890000	451000	1670000	2780000	916000	1310000	907000	1780000	1400000	1720000	810000	1830000	2040000	1060000	1400000	1320000	1050000
4260000	34600000	6980000	5550000	21700000	2390000	14600000	17700000	9810000	3430000	14500000	10600000	1750000	12900000	2510000	15900000	3330000	8900000
172000	436000	86497	174000	285000	68537	114000	174000	230000	377000	308000	165000	75803	243000	111000	291000	56206	141000
2540000	4170000	1430000	5440000	4070000	2520000	2250000	1410000	3620000	6110000	5310000	2870000	5250000	5800000	4000000	3780000	1720000	1300000
13999	17344	10368	32573	52669	15754	19765	12169	7310.6	6776.6	9429.2	1824	18694	31315	21379	40419	13243	24101
41934	53645	4317.9	27788	45307	38321	11185	5941.7	23250	25805	7997.5	4010.2	79300	15748	25422	5057	25074	9490.5
396000	500000	96355	475000	574000	462000	212000	104000	320000	375000	186000	124000	869000	283000	410000	139000	322000	197000
26934	23362	5944.6	28834	30015	36131	15066	7166.9	14825	29102	10745	13094	25430	26656	33774	20993	17062	19007
5640.1	13709	15698	15504	17730	13992	18136	19757	13622	7666.4	17865	10456	10418	14091	19444	20711	14910	16026
28055	23824	19741	34279	22185	29436	38076	32191	32708	25766	18645	22074	20218	54306	25531	26558	45434	25300
11675	50532	8970.2	43703	51630	29020	7159.2	95849	50421	14941	41010	8384.2	16332	74046	18981	43705	50390	37904
377000	511000	197000	647000	396000	502000	284000	131000	392000	397000	419000	315000	563000	384000	597000	487000	367000	277000
64635	285000	80227	115000	191000	94759	100000	46182	132000	83518	186000	96406	137000	138000	170000	146000	45069	62227
59271	140000	143000	59378	44745	21503	29479	12230	11765	106000	54240	88726	29451	34335	86395	15497	12569	9264.4
6633	3185.8	11021	20806	6501	11920	13139	2013.5	8153.6	6977.6	6966	4265.4	6173.5	9987.6	14959	15088	19289	8895.5
64118	437000	89320	1770000	2790000	637000	540000	768000	1180000	315000	323000	309000	789000	1050000	1680000	1440000	685000	960000

35565	266000	115000	139000	202000	75728	74478	78861	109000	113000	263000	84923	310000	98008	281000	149000	55229	99646
266000	177000	35821	254000	185000	201000	18667	82790	121000	421000	281000	178000	392000	127000	346000	96166	153000	157000
50805	22372	78393	133000	30315	82830	17138	33997	78498	44262	30750	28483	24841	32038	197000	35191	95246	58015
12689	15193	10945	24856	21060	14685	13699	4506.4	12520	12942	5913	14060	13945	22193	55745	11120	9765	12733
63114	60193	54610	111000	76215	148000	141000	111000	118000	101000	56670	61013	66421	178000	94091	104000	133000	152000
79902	101000	212000	256000	101000	248000	56544	91055	194000	84616	154000	62900	865000	77742	377000	153000	154000	97888
3140000	10900000	3690000	6040000	9180000	3000000	3680000	4010000	4370000	4790000	6840000	3780000	6410000	5370000	7140000	6020000	2370000	2910000
241000	298000	120000	430000	319000	180000	131000	52132	270000	354000	173000	160000	254000	429000	201000	379000	155000	127000
244000	1020000	320000	469000	821000	282000	403000	392000	684000	339000	659000	373000	445000	1350000	577000	706000	232000	393000
1150000	842000	36499	655000	622000	992000	122000	338000	122000	948000	770000	198000	542000	433000	191000	577000	578000	456000
483000	1030000	344000	531000	254000	210000	221000	81150	228000	1020000	558000	428000	528000	296000	393000	277000	159000	91629
3660000	5930000	1250000	6330000	5890000	5080000	4910000	1440000	5160000	3580000	2470000	3850000	3990000	6650000	6950000	6680000	1900000	2400000
237000	390000	57054	430000	502000	223000	215000	194000	334000	297000	261000	182000	334000	438000	259000	252000	222000	295000
68048	70524	48893	91759	116000	113000	65123	107000	83937	83720	61620	43808	140000	63672	72222	61156	52511	54860
291000	351000	90893	373000	374000	190000	189000	245000	350000	295000	295000	164000	211000	293000	288000	228000	52363	104000
28297	42086	17124	47743	32250	40722	63090	76153	35251	17517	24300	19906	44628	36078	44525	26915	40593	38815
35917	128000	28506	40943	59506	17447	10115	19611	32914	46129	54601	78371	50600	53284	23875	25103	25818	34255
149000	547000	228000	45874	174000	40082	381000	250000	70560	142000	390000	247000	50178	86352	12948	106000	135000	163000
149000	547000	228000	45874	174000	40082	381000	250000	70560	142000	390000	247000	50178	86352	12948	106000	135000	163000
159000	582000	300000	42986	264000	40214	113000	80152	95011	188000	563000	381000	42469	101000	21119	88532	137000	144000
202000	270000	249000	146000	297000	222000	314000	178000	253000	235000	200000	264000	120000	354000	186000	387000	371000	307000
28200000	33700000	5590000	36500000	34200000	17300000	19300000	20000000	29300000	25100000	26800000	7780000	17400000	35300000	39000000	40300000	22700000	36900000
470000	408000	129000	499000	268000	317000	182000	88138	420000	347000	232000	191000	274000	236000	383000	447000	158000	173000
142.0829346	313.9259693	193.7304397	183.863479	279.5888142	162.8872838	563.8959355	360.4696932	452.6608293	343.5157753	240.8970091	209.4408408	213.4528434	390.2127914	810.4741913	345.8105423	208.5174965	420.5825913
874.1419726	1729.935865	1020.491018	933.0043366	899.0642736	752.8080134	861.9807282	1283.637477	1279.416306	979.8337981	979.1175005	813.262326	1012.694464	916.9120566	942.4919964	817.1030589	988.6028069	1002.531171
448.9426965	434.2741121	688.0277203	688.8022426	548.3057568	408.5029112	3327.930211	600.4384724	920.9979091	554.22562	1072.932269	401.7015423	899.1126521	1883.660196	3758.243976	2916.979726	630.7144619	1313.787034
317.3099323	1.852355957	9.430511594	79.41934417	69.62962507	128.8162651	85.105636	57.27238151	86.58300302	20.74002063	11.74155348	26.02669703	11.28788628	32.9723815	42.57317695	19.38283356	182.8934803	146.0530905
1747.618053	132.006382	286.0941248	312.8210895	1349.434856	441.175113	1849.733838	363.5937985	299.9446885	327.0828947	61.95239297	126.7480013	80.22723594	1835.843171	48.36461314	302.6825883	696.2637611	242.6078571
165.1850309	171.9288626	101.2102622	328.1340489	250.1633426	360.4963045	273.4639639	215.8533875	343.2354225	320.4484347	69.56467877	153.0996362	283.6823434	183.9789603	96.86537701	103.4439158	128.5278913	46.8173384
1058.280155	236.9545139	329.2529095	790.2353884	341.3470069	638.7837691	176.2344591	344.0641084	858.1065855	529.9271903	192.2288949	575.1154266	1263.456718	1078.64705	532.366478	371.1182514	584.7998629	607.2257006
608.8051762	845.0674001	881.6554316	881.3443269	925.0590975	696.0939695	807.3844564	899.2017537	1127.125058	562.0300949	717.937842	619.3112365	720.0268653	1336.371154	603.7552479	1486.23564	530.6094724	749.5810821
179.6520063	498.2295283	43.00369112	1.16261545	293.1843408	32.96784283	51.98347426	15.95090829	415.4456131	1428.226243	38.79380517	10.61319487	249.9091221	160.736789	77.14950581	2.899055922	12.67702343	2.699998632
4697.83103	4160.980032	3325.78034	5778.637738	5285.278977	4895.364049	9146.668012	7396.655478	6273.383191	4610.496076	5394.244499	5099.313745	3256.317319	11235.70067	2987.396624	10796.63097	15098.5994	6678.051704
20.04935014	0	0	22.04622853	0	76.73891235	74.00874789	0	19.52060592	5.423247685	0	22.47229991	7.80231675	88.54662822	0	67.98955337	0	27.69899387
8.176151005	21.20240173	1.676888179	20.69827121	18.47337505	70.56324896	48.65179079	2.891003751	64.39285543	38.08922947	47.565751	35.92288941	8.227280933	53.88637269	12.39645386	77.89942773	48.08530289	22.56614287
72.43390557	95.19340883	93.72377228	10.02254019	490.7940024	132.6171989	23.11554889	26.31267355	7.327262276	98.77659457	21.87228178	6.032616714	305.5698675	120.4839196	7.848106442	24.31950802	19.9531555	17.08933613
227.1437552	620.7308826	519.1164057	97.60781655	1550.562854	477.1672342	36.20160995	35.62780388	136.5883903	148.3882575	39.76064449	75.87730996	828.0682486	377.9580928	411.8470747	54.89692534	216.7951052	58.77569136
1321.118852	1377.72781	4147.054677	1332.094222	1611.392499	1589.191066	1661.971562	1641.373859	1637.182208	1366.311385	1916.483818	1545.135143	976.9571205	1683.730618	1487.064377	4157.796473	1611.014174	1314.27751
205.603912	25.58978253	18.84856286	18.26019298	6.239464288	85.03544741	0	2.94706E-07	39.45443465	45.68603536	17.77409366	6.375910002	7.636288933	0	29.40594761	6.302428962	6.954860406	94.50892555
1719.040219	20.47843574	14.55635062	300.8841064	208.0289952	434.8244268	282.4833244	361.5439172	297.1607311	21.66802464	6.534080243	1.64622383	130.3419721	65.63976818	9.197011566	0.189087898	1028.365917	1048.735084
171.0236896	191.7480911	220.8766488	120.7746759	121.2019893	89.05697668	172.7908446	244.628652	228.7409459	192.1668045	91.22995802	162.0265817	25.57330203	81.89862464	75.21322766	380.2832403	134.7583382	66.04071715
43.83336307	127.0668306	54.24095728	87.114366	105.9137204	61.13712243	92.87424869	34.3750917	85.10194459	34.97808044	185.1055421	74.79909161	74.68177693	109.8295824	139.5481467	101.0279407	49.57801529	52.30139593
34.95128904	39.6373623	25.58130913	39.77584098	38.86643581	51.34846117	55.87948581	44.55234807	84.20776204	28.75715129	26.9327377	27.7815883	37.24923972	98.6269746	9.255152334	87.98118513	20.79785368	42.47053392
27.33905486	10.46581918	9.429319918	19.26462157	21.80039535	143.2239735	45.1972948	89.24414599	171.656575	118.1096156	38.5752764	71.70511188	46.03723639	237.5128018	31.76795108	17.42327773	38.76257172	42.85444517
9.042824719	20.74796069	8.934365952	10.48763966	14.46578984	54.79979068	18.223074	26.93234068	45.35492366	34.42035461	11.33094431	39.24027146	42.80866978	91.23148899	35.48884384	22.90739289	31.39334768	31.98293356
